# Identification and Validation of Immune- and Stemness-Related Prognostic Signature of Melanoma

**DOI:** 10.3389/fcell.2021.755284

**Published:** 2021-11-05

**Authors:** Yan Zhang, Jing Peng, Heng Du, Niannian Zhang, Xianfeng Fang

**Affiliations:** ^1^Department of Dermatology, The First College of Clinical Medical Sciences, China Three Gorges University, Yichang, China; ^2^Department of Dermatology, Yichang Central People’s Hospital, Yichang, China

**Keywords:** melanoma, overall survival, tumor immune microenvironment (TME), tumor stem cell, prognostic signature

## Abstract

**Purpose:** Our aim was to construct a signature that accurately predicted the prognostic and immune response of melanoma.

**Methods:** First, the weighted co-expression network analysis (WGCNA) algorithm was used to identify the hub genes related to clinical phenotypes of melanoma in the cancer genome atlas (TCGA) database. Nest, the least absolute shrinkage and selection operator (LASSO) analysis was used to dimensionality reduction of these hub genes and constructed a prognostic signature to predict the prognosis and immunosuppressive response of melanoma.

**Result:** Through in-depth analysis, we constructed a 5-mRNA prognostic signature and verified its prognostic value in internal (TCGA-SKCM, *n* = 452) and external independent datasets (GSE53118, *n* = 79). Based on this signature, the tumor immune microenvironment (TME) of melanoma was characterized, and the result was found that patients in the high-risk group had lower CD8 T cell infiltration and immune checkpoint expression (PD-1, PD-L1, CTLA4), as well as higher M0/M2 macrophage infiltration. Our results also found the risk score based on a 5-mRNA signature was significantly associated with tumor mutational burden (TMB) and tumor stem cell markers (CD20, CD38, ABCB5, CD44, etc.). Lastly, we built a nomogram for clinician prediction for the prognosis of patients with melanoma.

**Conclusion:** Our findings indicated that the 5-mRNA signature has an important predictive value for the overall survival of melanoma. By analyzing the tumor immune microenvironment and tumor stem cell marker between different groups, a new method is provided for the stratified diagnosis and treatment of melanoma.

## Introduction

Melanoma is one of the most aggressive and fatal forms of skin tumor, although it accounts for about 4% of all skin cancers, its mortality rate is as high as 80% (Kim et al., 2019). Melanoma can invade within any tissue containing melanocytes, especially in the skin (Guo et al., 2016). Surgery, radiation therapy, and chemotherapy are common treatments for melanoma. For patients with primary melanoma, the 5-year survival rate can reach up to 95%. However, for patients with metastatic melanoma, the 5-year survival rate is less than 10% due to the high rate of recurrence and lack of efficient biomarkers (Schadendorf et al., 2018). Fortunately, the molecular characterizations of melanoma have achieved considerable advances. Current research shows that BRAF, NRAS, and C-Kit genes are closely related to the pathogenesis of melanoma (Ponti et al., 2017). As an emerging treatment in recent years, PD-1 has been shown to significantly improve the survival rate of melanoma patients. The identification of effective biomarkers that can estimate responses to immunotherapy for melanoma has become a new trend of research. Melanoma with BRAF mutations appears to benefit from targeted BRAF and MEK therapy. Moreover, PD-L1 can predict which patients will benefit from targeting CTLA-4 (Ponti et al., 2017).

In this study, by constructing a WGCNA network, we grouped some genes with the same characteristics into the same cluster. Next, we calculated the correlations of all clusters with some important clinical phenotype data. Then, after a battery of screening tests, we developed and used independent external data to validate a 5-mRNA signature that predicted melanoma prognosis. Univariate and multivariate Cox regression analyses combined with key clinical characteristics were used to construct a graph that clinicians can use to predict the prognosis of melanoma. Finally, we found significant associations between risk scores and melanoma immune microenvironment and tumor stem cell markers.

## Materials and Methods

### Data Collection and Data Preprocessing

**FIGURE 1:**
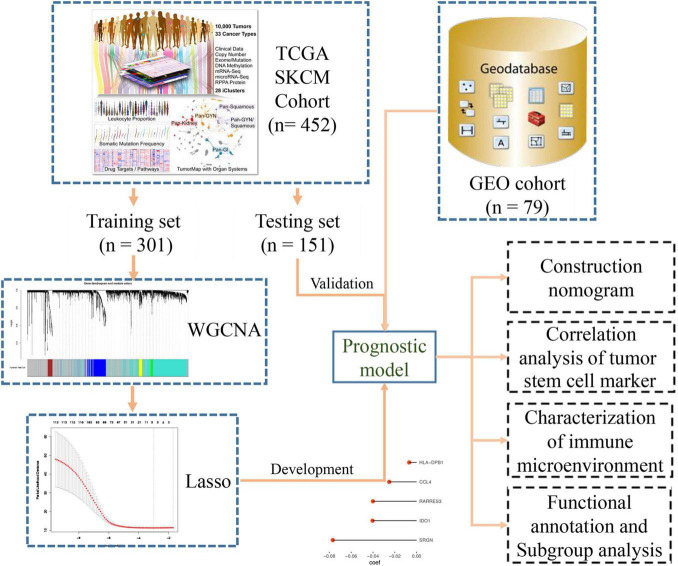
Flow diagram of the study.

Transcriptome (*n* = 472) and clinical feature data (*n* = 477) were downloaded from the UCSC dataset.^1^ The RNA-seq data included 471 melanoma samples and 1 normal skin sample. The clinical information of melanoma samples included survival events, tumor staging, grade, Breslow depth value, and Clark level value. We selected samples (*n* = 472) with both RNA-seq data and clinical phenotype data for subsequent analysis. Later, when building the Lasso regression model, we only retained samples (*n* = 452) containing both RNA-seq data and survival data for analysis. We installed a 2:1 ratio to randomly group these samples as the training set (*n* = 301) and internal validation set (*n* = 151), respectively. All the data used for survival analysis comes from an attachment to an article (Liu et al., 2018). The somatic mutation data (MuTect2 Variant Aggregation and Masking) were also downloaded from this website. We built a flow chart for the whole process of the experiment (Figure 1). We converted our downloaded RNA-seq data (FPKM values) into TPM values, which makes the samples more comparable (Wagner et al., 2012). To filter out genes whose expression has not changed much, we then selected the genes in the top 25% (*n* = 4,939) of the variance for subsequent analysis. For the processing of externally validated data from the GEO database, we download public data from the GEO database, then RMA corrected, standardized, and then logarithmic.

### Establishment of a Co-expression Network

We used those genes from one step to build a co-expression network by the R package “WGCNA” (Langfelder and Horvath, 2008). First, the Pearson correlation coefficient between samples is calculated to remove abnormal samples (Supplementary Figure 1). Then we constructed an adjacency matrix through the use of Pearson’s correlation analysis for all pairs of genes. Finally, by associating the modules with the clinical features, we selected the modules most relevant to the clinical features of greatest concern for subsequent analysis. We defined the cor.geneModuleMembership > 0.8 and cor.geneTraitSignificance > 0.2 as the threshold for screening hub genes in a module.

### Functional Annotation

We uploaded all the genes of the blue module to DAVID’s website^2^ for GO analysis and KEGG analysis. In order to understand the enrichment of signal pathways in the classification of the 5-mRNA signature, the gene set enrichment analysis (GSEA) was carried out with R package “ClusterProfiler.”

### Immune Infiltration and Tumor Stem Cell Related Analysis

The mutation frequency less than 0.05 and synonymous mutations were removed. We used CIBERSORT (Newman et al., 2015) online software to quantify the immune infiltration of BLCA-SKCM based on deconvolution algorithm. The LM-22 gene signature which included 22 types of human immune cell was used as the set of reference gene-expression values to perform immune infiltration analysis. The R package “estimate” (Yoshihara et al., 2013) was used to quantify the stromal and immune cell admixture, stromal cells and immune cells were the main components of normal cells in tumor tissue. The association between tumor stem cell markers (CD20, ABCB5, CD38, etc.) and risk score was calculated using Pearson’s correlation analysis.

### Statistical Analysis

We use R package “survival” for univariate and multivariate analysis, R package “forestplot” for forest map, and then R package “rms” for the nomogram. The calibration curve and the decision curve analysis performed by R package “rmda” were used to measure the accuracy of the nomogram. The statistical test method of the two groups of data involved in this paper was the *t*-test, which used the two-sample *t*-test and the Welch two-sample *t*-test, respectively, according to whether the variance was homogeneous. The statistical method used for all survival analyses was the Log-rank test.

## Results

### Co-expression Network Analysis

The R package of “WGCNA” is used to perform co-expression network analysis. The Pearson correlation analysis was used to detect the outlier samples and we found 8 samples were outlier samples, then we removed them (Supplementary Figure 1). The 4,939 genes with the first 25% variance were put with similar expression patterns into modules by cluster analysis. The power of β = 10 was chosen for the soft-thresholding to develop a scale-free network (scale-free *R*^2^ = 0.91, Supplementary Figure 2).

**FIGURE 2:**
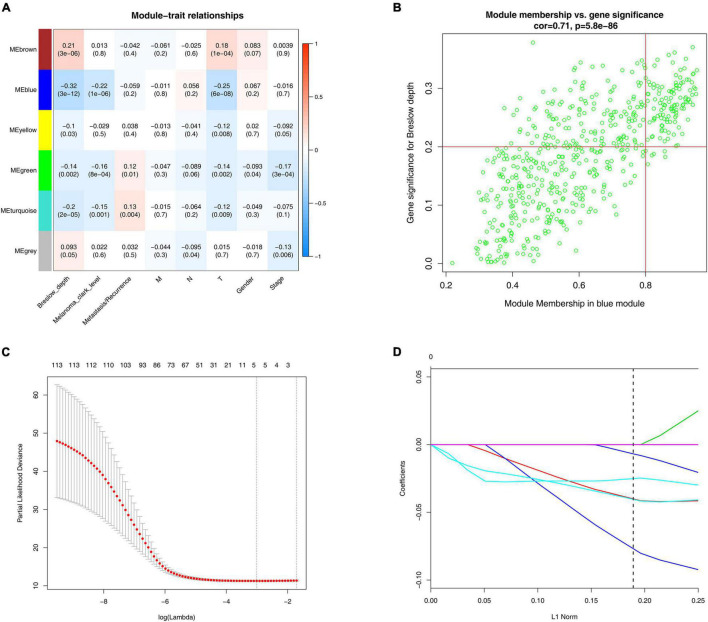
WGCNA and LASSO analysis. **(A)** Identification of modules associated with the clinical traits of melanoma. Heatmap of the correlation between module eigengenes and clinical traits of melanoma. **(B)** Scatter plot of module eigengenes related to Breslow depth in the blue module. **(C)** Tenfold cross-validation for parameter chosen. Here, λ = 0.049 was chosen by 10-fold cross-validation. **(D)** Construction of prognosis signature based on the LASSO algorithm.

We finally got six modules, among which the blue module showed a strong negative correlation with the Breslow depth (*r* = −0.32, *p* = 3e-12). It also showed a strong negative correlation with the Clark level (*r* = −0.22, *p* = 1e-06) and the T stage (*r* = −0.25, *p* = 6e-08, Figure 2A). So, we finally chose the blue module as our focus, and then finally we got 116 hub genes (Figure 2B).

### Establishment and Validation of the Signature

**FIGURE 3:**
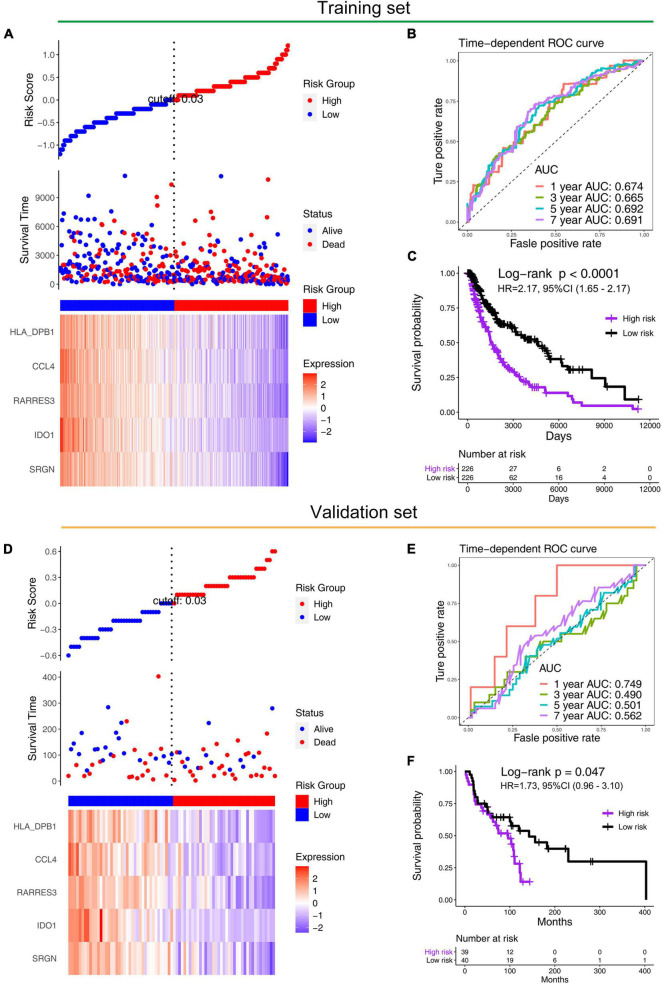
Performance of prognostic models in training (TCGA) and validation sets (GSE53118). Distribution of the risk scores (the upper panels), distribution of prognostic status (the middle panels), and distribution of 5-gene expression in the **(A)** training set and **(D)** validation set (the lower panels). The time-dependent ROC curve of prognostic model in the **(B)** training set and the **(E)** validation set. The Kaplan–Meier survival in the **(C)** training set and the **(F)** validation set.

We constructed a prognosis signature based on 5-mRNA through LASSO algorithm (Figures 2C,D). In the training set (HR = 2.69, 95%CI: 1.94–3.71, *p* < 0.0001) and the external validation set (HR = 1.73, 95%CI: 0.96–3.10, *p* = 0.047), the prognostic model showed high predictive power. The risk score = −0.08 × SRGN expression value—0.04 × IDO1 expression value—0.04 × RARRES3 expression value—0.03 × CCL4 expression value—0.007 × HLA-DPB1 expression value. We grouped the population according to the median risk score. We found that the risk of death in the training set and the validation set increased significantly with the increase of the risk score (Figures 3A,D, the middle panels), and the genes used to construct the 5-mRNA prognosis signature decreased with the increase of the risk score (Figures 3A,D, the lower panels). The time-dependent ROC curve showed that the AUC of 1-, 3-, 5-, and 7-years was mostly greater than 0.65 (Figures 3B,E). Survival analysis also showed good predictive value of the signature; the high-risk group tended to have a worse prognosis (Figures 3C,F).

**FIGURE 4:**
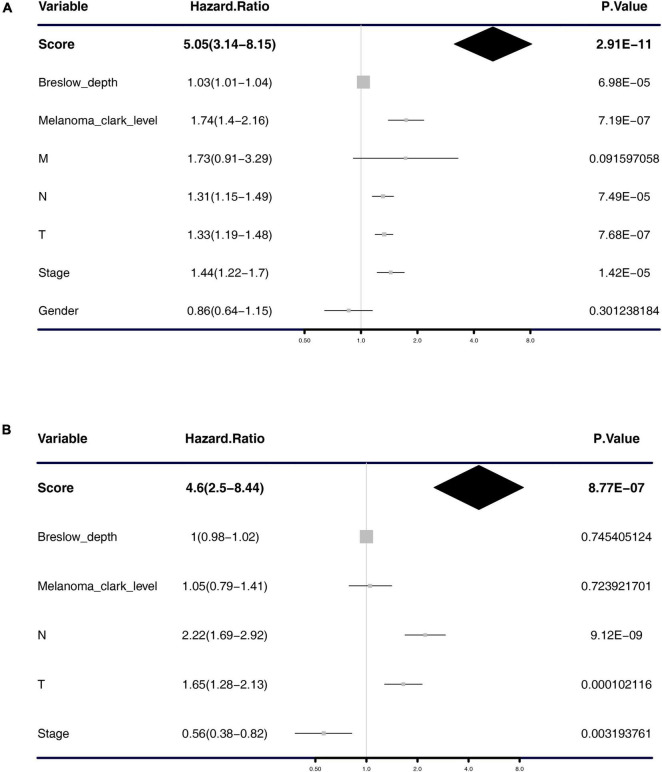
Forest plot of univariate and multivariate Cox regression analysis. Univariate analysis **(A)** and multivariate analysis **(B)** of the risk scores, Breslow depth, TNM classification, stage, etc.

Univariate and multivariate Cox regression analyses were performed to test the independence of the predictive power of risk scores. First, the signature risk score, TMN stage, gender, histologic stage, Breslow depth, and Clark level were performed with the univariate analysis (Figure 4A and Table 1), we found that risk score (HR = 5.05, *p* = 2.91e-11), Breslow depth (HR = 1.03, *p* = 6.98e-05), Clark level (HR = 1.74, *p* = 7.19e-07), T stage (HR = 1.33, *p* = 7.68e-07), N stage (HR = 1.31, *p* = 7.49e-05), and pathological stage (HR = 1.44, *p* = 1.42e-05) showed good predictive power of prognosis. Next, we put this factor to perform the multivariate analysis. The results showed that risk score was an independent predictor of melanoma risk (HR = 4.6, *p* = 8.77e-07, Figure 4B and Table 1). The subgroup analysis’s results exhibit the 5-mRNA signature showed good predictive value among different tumor stages and grades (Supplementary Figure 3). We also used the GEPIA database (Tang et al., 2017)^3^ to measure the prognostic value of the 5 genes in the signature. The results showed that all the 5 genes were protective factors for the prognosis of melanoma (Supplementary Figure 4).

**TABLE 1:** Univariate analysis and multivariate analysis of the immune-related signature.

**Variable**	**Univariate analysis**			**Multivariate analysis**		
	**HR**	**95%CI**	***P***	**HR**	**95%CI**	***P***
Risk score	5.05	(3.14−8.15)	<0.001	4.6	(2.5−8.44)	<0.001
Breslow depth	1.03	(1.01−1.04)	<0.001	1	(0.98−1.02)	0.745
Clark level	1.74	(1.4−2.16)	<0.001	1.05	(0.79−1.41)	0.724
M	1.73	(0.91−3.29)	0.092			
N	1.31	(1.15−1.49)	<0.001	2.22	(1.69−2.92)	<0.001
T	1.33	(1.19−1.48)	<0.001	1.65	(1.28−2.13)	<0.001
Stage	1.44	(1.22−1.7)	<0.001	0.56	(0.38−0.82)	0.003
Gender	0.86	(0.64−1.15)	0.301			

### The Construction of the Nomogram and Its Accuracy Verification

**FIGURE 5:**
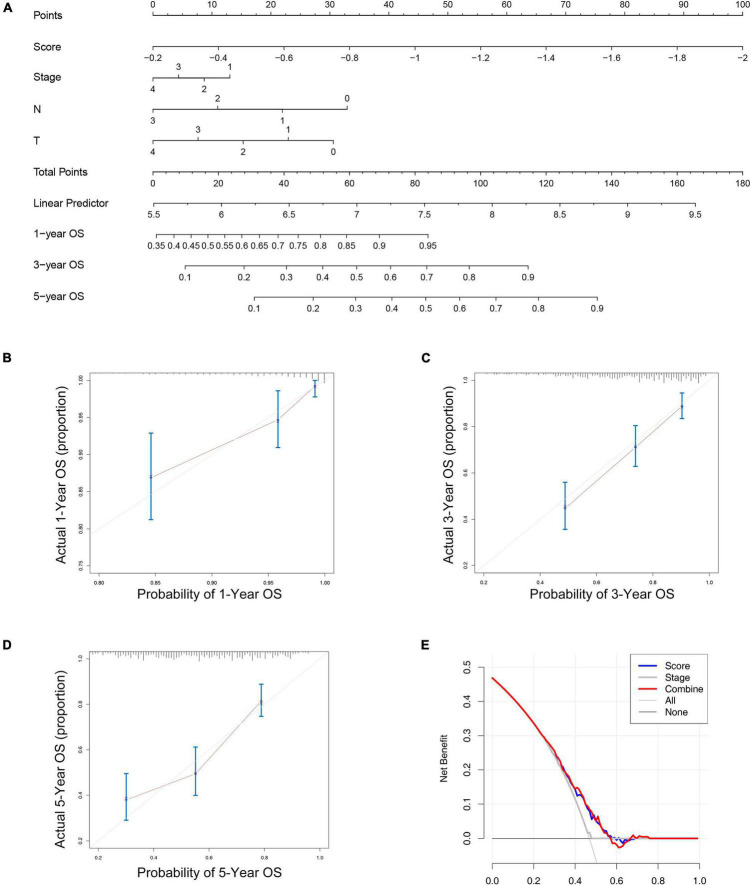
The nomogram to predict the overall survival of melanoma. **(A)** The nomograms for predicting the proportion of melanoma patients with 1-, 3-, or 5-year overall survival. Plots depict the calibration of the nomogram between predicted and observed 1- **(B)**, 3- **(C),** or 5- **(D)** year outcomes. **(E)** The DCA indicated the clinical utility of the nomogram was well, the risk score had a better net benefit compared to tumor stage, and the combination of the two had a better net benefit than either of the two.

From the results of the previous step, we constructed a nomogram combining the risk score and important clinical characteristics (Figure 5A). The calibration curve showed that the predictive power of the nomogram at 1, 3, and 5 years was accurate (Figures 5B–D). The DCA indicated the clinical utility of the nomogram was well, the risk score had a better net benefit compared to tumor stage, and the combination of the two had a better net benefit than either of the two (Figure 5E).

### Functional Annotation and Gene Set Enrichment Analysis

We uploaded all the genes of the blue module to DAVID’s website to perform GO and KEGG analysis, and the results of KEGG analysis indicated that the “Staphylococcus aureus infection,” “Antigen processing and presentation,” “Graft-vs.-host disease” were enriched in the blue module (Supplementary Figure 5D and Supplementary Table 1). The GO analysis showed that the blue module was related to “immune response,” “type I interferon signaling pathway,” “inflammatory response,” “T cell receptor signaling pathway,” “extracellular exosome,” “peptide antigen binding,” “MHC class II receptor activity,” and so on (Supplementary Figures 5A–C and Supplementary Table 1). These results indicated that the genes of the blue module were significantly related to the immune process. Then we explored the difference in signaling pathways between the high- and low-risk groups by GSEA under the threshold of p.adjust < 0.05. The high-risk groups were mainly enriched in “MYC targets v2” related pathways, while the low-risk groups were mainly enriched in “ALLOGRAFT REJECTION,” “INTERFERON_ALPHA_RESPONSE,” “INFLAMMATORY_RESPONSE,” and so on (Supplementary Figure 6C and Table 2). Through GO and KEGG analysis of risk scores, we found that risk scores were significantly correlated with T cell activation, regulation of lymphocyte activation, and regulation of T cell activation functions, and were correlated with cell adhesion molecules (CAMs), allograft rejection, antigen processing and presentation and so on pathways (Supplementary Figures 6A,B and Supplementary Table 2).

**TABLE 2:** Gene set enrichment analysis (GSEA) of the prognostic signature.

**Term**	**setSize**	**NES**	***p*-value**	**p.adjust**
MYC_TARGETS_V2	38	4.40	<0.001	0.01
COAGULATION	88	−1.51	0.004	0.015
APOPTOSIS	115	−1.91	<0.001	0.01
KRAS_SIGNALING_UP	145	−2.08	<0.001	0.01
IL2_STAT5_SIGNALING	149	−2.18	<0.001	0.01
COMPLEMENT	162	−2.28	<0.001	0.01
TNFA_SIGNALING_VIA_NFKB	164	−2.29	<0.001	0.01
IL6_JAK_STAT3_SIGNALING	79	−2.59	<0.001	0.01
INFLAMMATORY_RESPONSE	166	−2.69	<0.001	0.01
INTERFERON_ALPHA_RESPONSE	92	−2.97	<0.001	0.01
ALLOGRAFT_REJECTION	177	−2.99	<0.001	0.02

*NES, Normalized enrichment score.*

### Correlation Analysis of Risk Score With Tumor Immunity and Tumor Stem Cells

**FIGURE 6:**
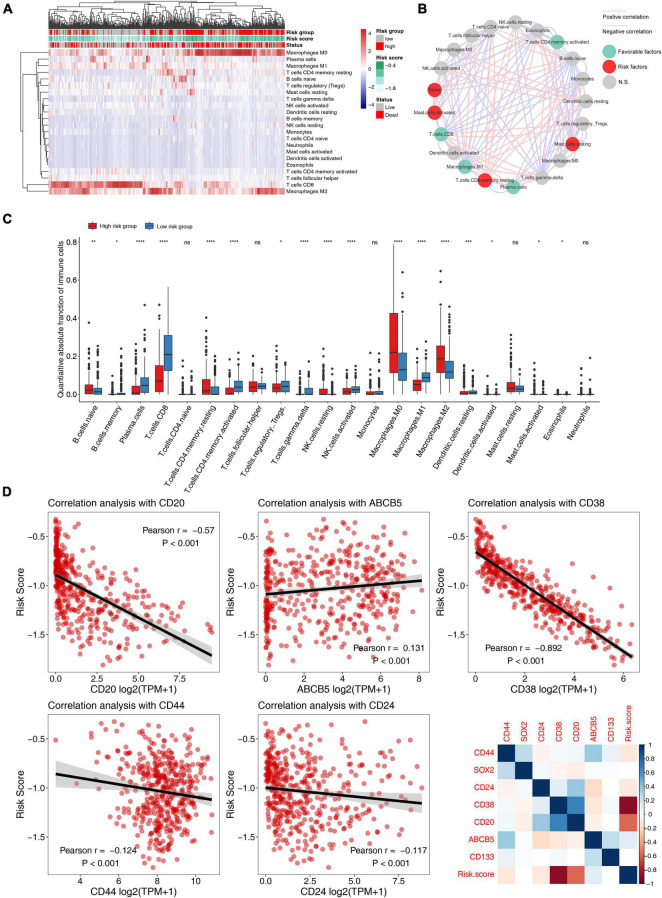
Correlation analysis of 5-mRNA prognostic signature with tumor immune microenvironment and tumor stemless in melanoma. **(A)** The heatmap of immune cell infiltration based on the 5-mRNA signature. Red squares indicate high levels, while blue squares indicate low levels. **(B)** Cellular interaction of the tumor immune microenvironment cell types and risk scores. The red line represents a significant positive correlation, and the blue line represents a significant negative correlation. The red dots indicate that it is a risk factor for melanoma prognosis, the green dots indicate a protective factor, and the gray dots indicate that it is not significantly predictive of prognosis. **(C)** Distribution of 22 types of immune-infiltrating cells in different risk groups. **(D)** Scatter plot and heatmap of correlation analysis between risk score and tumor stem cell markers. *, *p* < 0.05; **, *p* < 0.01; ***, *p* < 0.001; ****, *p* < 0.0001; *ns*, no significant.

Through the functional annotation in the previous step, the results exhibit that the low-risk group had a significant relationship with tumor immune-related pathways, so we then analyzed the characteristics of this prognostic model in the immune microenvironment (Figures 6A,B). We quantified 22 major immune cells using CIBERSORT software and found that the main immune infiltration cells in melanoma are CD8 T cells and macrophages (Figures 6A,D and Supplementary Figures 7A–C), the low-risk group had higher CD8 T cell and macrophages M1 infiltration and lower macrophage M0/2 infiltration (Figure 6C). It was also found that these recognized immune checkpoints (PD-L1, PD-L1, CTLA4, etc.) were highly expressed in the low-risk group (Supplementary Figures 7E–G). Our study found that in patients with high TMB or BRAF mutation, the risk score was low (Supplementary Figures 7D,H). By calculating risk score and the correlation of the tumor stem cell marker, results showed that the risk score significantly associated with melanoma cancer stem cell markers (CD20, ABCB5), not only such, risk score also some correlation with other tumor markers, which suggests that tumor stem cell factors contributed to the prognosis of different risk groups for different reasons (Figure 6D).

## Discussion

Melanoma is one of the most aggressive forms of skin cancer worldwide, with high mortality rates. Every year, more than 50,000 people die of melanoma (Sahranavardfard et al., 2019). The melanocytes transform into melanoma is a complex process that requires many factors, and the immune-related factors play an important role in this process (Schadendorf et al., 2018). The overall survival rates for metastatic melanoma have increased due to immunotherapy being widely used, but the efficacy of immunotherapy varied widely among different populations. Therefore, it is important to search meaningful molecular biomarkers to determine the progress and prognosis of melanoma, and these molecular biomarkers can not only represent favorable or poor prognosis but also be used to help patients choose appropriate immunotherapy.

Since stemness markers of melanoma have been proved to be significantly correlated with immune microenvironment, for example, CD20 is also a surface marker of B cells (Lee et al., 2021), we also explored the correlation between 5-mRNA prognostic signature and some stemness markers, and found a significant correlation between the two. Over the years, several tumor stem cell markers associated with melanoma have been gradually identified (Marzagalli et al., 2019). For example, a marker known as ABCB5 has been found to be significantly associated with melanoma drug resistance. For another example, CD20 is a recognized marker of melanoma stem cell surface, and there have been quite a few studies targeting CD20 for the treatment of melanoma (Schlaak et al., 2012; Song et al., 2015; Zeng et al., 2018; Marzagalli et al., 2019). We found that 5-mRNA prognostic signature was also significantly associated with some stemness markers in other tumors (stemness marker of breast cancer and pancreatic cancer: CD44/CD24). Studies have found that the high CD38 in melanoma patients can be used as biomarkers for anti-PD-1 resistance (Plesca et al., 2020). The 5-mRNA prognostic signature we constructed and validated was significantly associated with the two melanoma stem cell markers, suggesting that underlying stemness factors influence the prognosis and immune microenvironment of melanoma patients.

Many reports have applied the WGCNA algorithm in cancers to explore potential biomarkers of prognosis (Tang et al., 2018; Chen et al., 2019; Dong et al., 2019; Wang et al., 2019; Yang L. et al., 2019; Zhao et al., 2019), but there are not very many similar studies on melanoma. Yang L. et al. (2019) identified the expression of three genes (STK26, KCNT2, CASP12) was correlated with the prognosis of skin cutaneous melanoma (SKCM) using the WGCNA algorithm and LASSO Cox regression model. Another study used network-based co-expression analysis to find 24 hub genes were involved in the immune response and development of metastatic melanoma, and those hub genes can be used as biomarkers for the early diagnosis of melanoma (Wang et al., 2018).

In our study, a total of 6 co-expression modules were identified using the WGCNA method. We found the blue module showed a negative correlation with the Breslow depth, Clark level, and T stage. The Breslow depth has been found to be associated with progressive migration of CD1a^+^ DC subsets in early melanoma (van den Hout et al., 2017). This suggests that the Breslow depth is closely related to the immune microenvironment of melanoma. By LASSO Cox regression analysis, we constructed a prognosis model based on 5-mRNA (SRGN, IDO1, RARRES3, CCL4, HLA-DPB1), which has great predictive value for the overall survival of melanoma. Through GO and KEGG analysis we found the blue module was significantly related to the immune process, such as antigen processing and presentation, immune response, type I interferon signaling pathway, T cell receptor signaling pathway, etc. In addition, GSEA showed that the low-risk groups were mainly enriched in “T CELL RECEPTOR SIGNALING PATHWAY,” “B CELL RECEPTOR SIGNALING PATHWAY,” “JAK STAT SIGNALING PATHWAY,” etc. indicating that the patients in the low-risk group had a stronger immune response than those in the high-risk group. Then, we quantified 22 major immune cells using CIBERSORT software and found that the low-risk group had higher CD8 T cell infiltration and lower macrophage infiltration. CD8 T lymphocytes play an important role in immunity against tumors. Some studies found that the infiltration of CD8 T cells in melanoma has been associated with longer survival in patients (Edwards et al., 2018; Yang S. et al., 2019), this was consistent with our results. Moreover, increasing CD8 T cell infiltration could enhance melanoma radiosensitivity (Chen et al., 2018). These results indicated that the 5-mRNA prognostic signature could predict melanoma for the benefit of immunotherapy.

The five hub genes were related to the immunity of melanoma. In detail, IDO1 (Indoleamine 2,3-Dioxygenase 1) was a monomeric heme-containing oxidoreductase that catalyzes the rate-limiting step of tryptophan (Trp) metabolism to kynurenine (Kyn) (Jung et al., 2019). IDO1 suppresses the immune response in T cells by depleting Trp and accumulating Kyn in the local tumor microenvironment (Xu et al., 2018). IDO1 overexpresses in many types of human malignancies, such as melanoma (Brody et al., 2009). CCL4 (C-C Motif Chemokine Ligand 4) is a chemokine currently thought to mediate the recruitment of CD8^+^ T cells and regulatory T cells (Jamal et al., 2017). In general, increased tumor immune infiltration significantly improves survival in melanoma patients (Jacquelot et al., 2018), therefore CCL4 was the potential predictive biomarker most likely to respond to immunotherapy of melanoma. In addition, the role of chemokines in evaluating the prognosis of melanoma patients may be a new trend of research.

In conclusion, through deep mining, we have built a 5-mRNA prognostic signature that not only predicts the overall survival rate of melanoma but also according to its classification of immune microenvironments, it is possible to predict the effect of immunotherapy.

## Data Availability Statement

The datasets presented in this study can be found in online repositories. The names of the repository/repositories and accession number(s) can be found in the article/Supplementary Material.

## Author Contributions

YZ, JP, and HD performed the data management and analysis procedures. YZ, HD, and XF analyzed the results. YZ, NZ, and JP contributed to the writing of the manuscript. All authors contributed to the study conception and design, read and approved the final manuscript.

## Conflict of Interest

The authors declare that the research was conducted in the absence of any commercial or financial relationships that could be construed as a potential conflict of interest.

## Publisher’s Note

All claims expressed in this article are solely those of the authors and do not necessarily represent those of their affiliated organizations, or those of the publisher, the editors and the reviewers. Any product that may be evaluated in this article, or claim that may be made by its manufacturer, is not guaranteed or endorsed by the publisher.

## Footnotes

1
https://gdc.xenahubs.net

2
https://david.ncifcrf.gov/

3
http://gepia.cancer-pku.cn/
