# Supplementary material for: Identification and Validation of Immune- and Stemness-Related Prognostic Signature of Melanoma
Source: Front Cell Dev Biol. 2021 Nov 5;9:755284. doi: 10.3389/fcell.2021.755284 (PMC8602573; doi:10.3389/fcell.2021.755284)
Supplement: Supplementary file 1 [file Data_Sheet_1.docx]

**Identification and validation an immune- and stemness-related prognostic signature of melanoma**

**Supplementary figures**


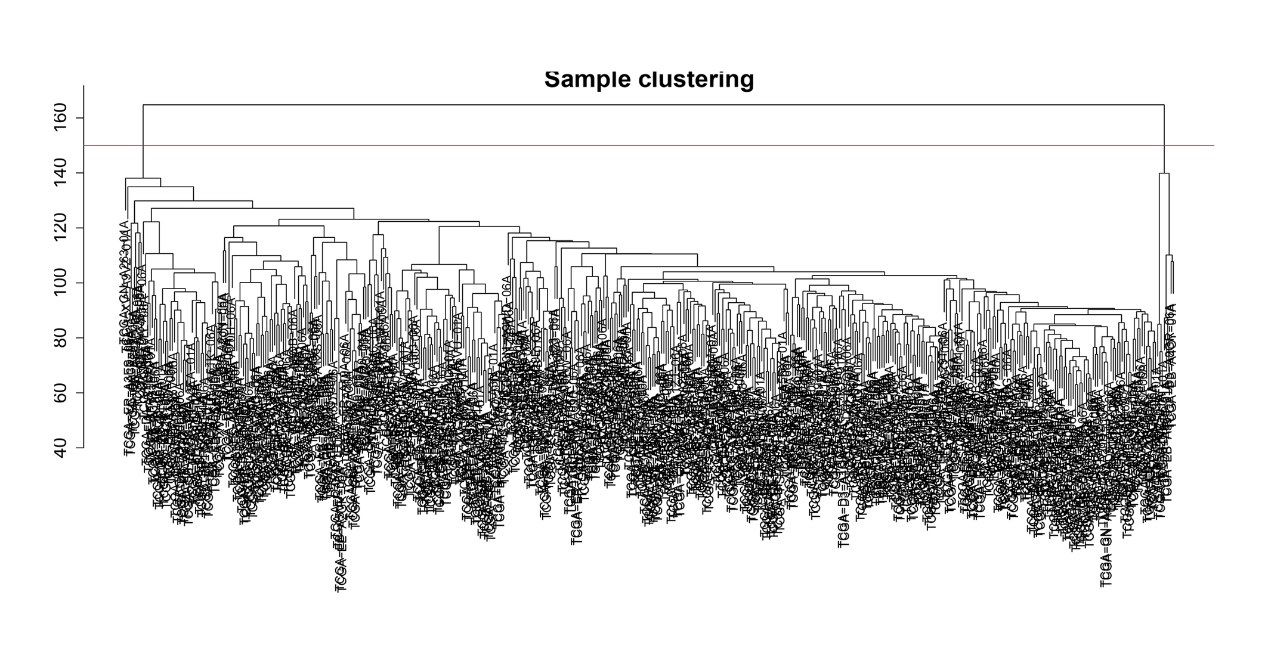


**Supplementary Figure S1.** **Cluster dendrogram, samples clustering to detect outliers.**


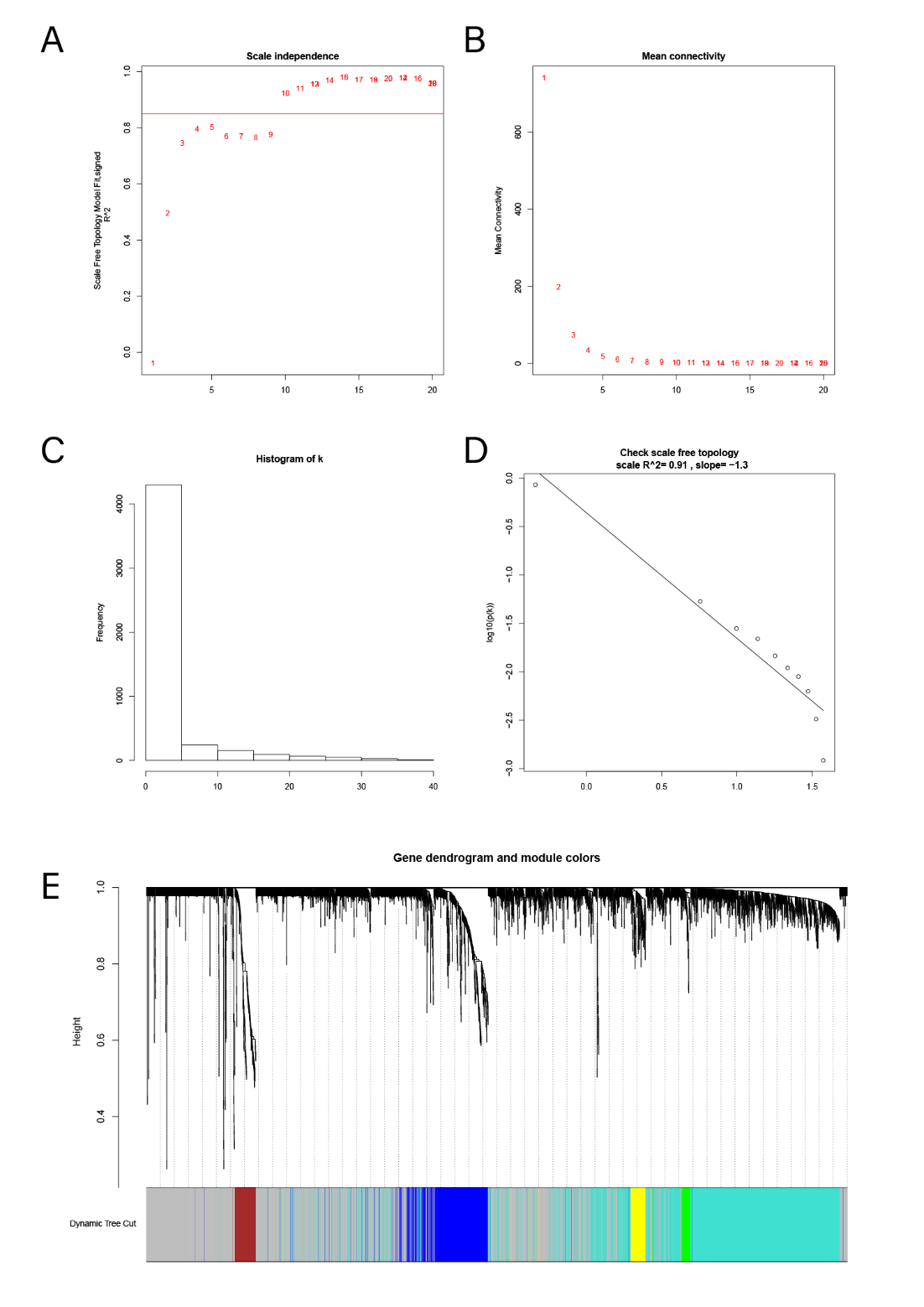


**Supplementary Figure S2. Determination of soft-thresholding power in the weighted gene co-expression network analysis (WGCNA).** **(A)** Analysis of the scale-free fit index for various soft-thresholding powers (β). **(B)** Analysis of the mean connectivity for various soft-thresholding powers. **(C)** Histogram of connectivity distribution when β = 10. **(D)** Checking the scale free topology when β = 10. **(E)** Dendrogram of all differentially expressed genes clustered based on a dissimilarity measure (1-TOM).


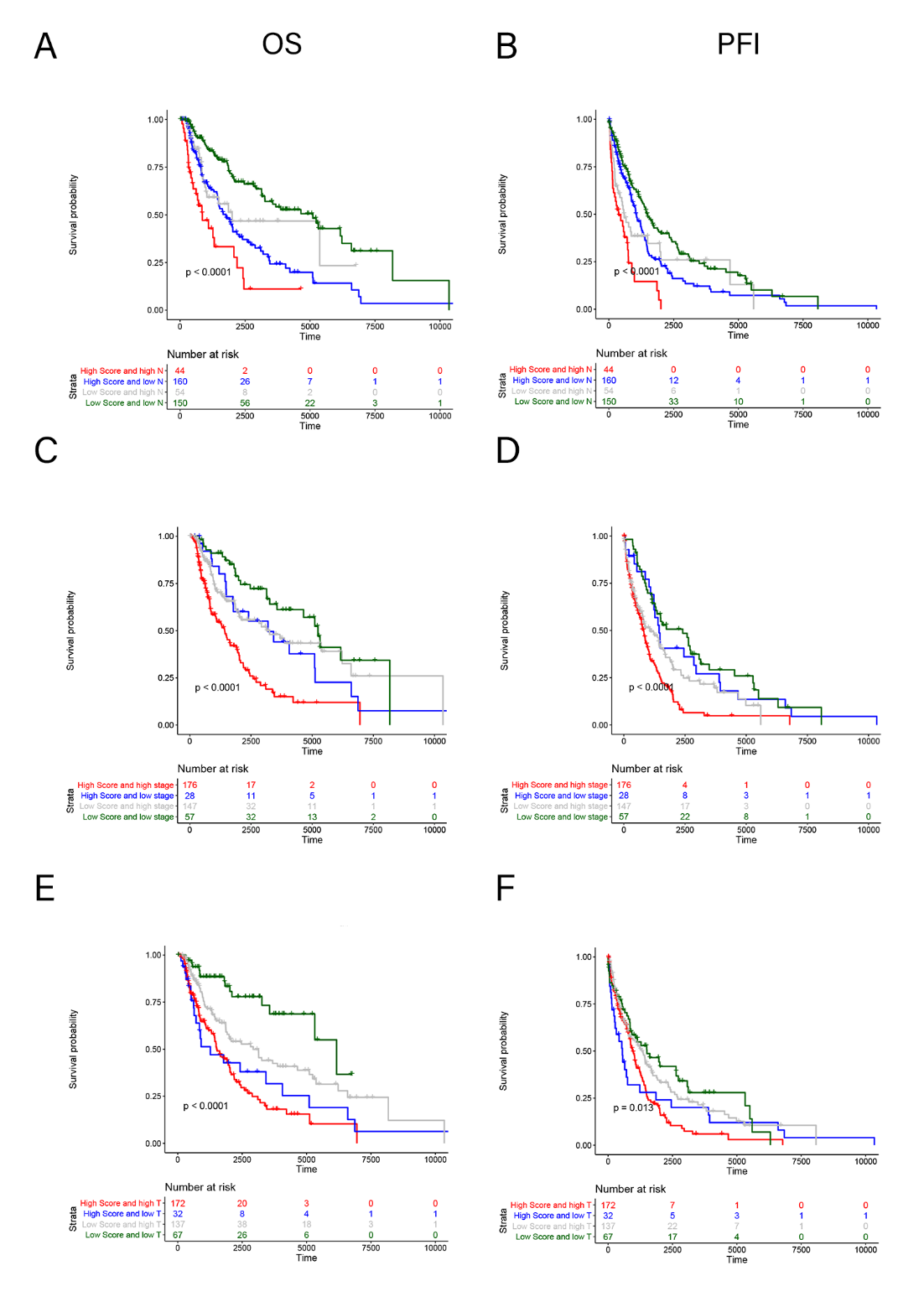


**Supplementary Figure S3.** **Subgroup survival analysis of 5-mRNA signature.** Overall survival and Progression-free interval analyses of 5-mRNA signature were performed in different N stages **(A-B)**, Stage **(C-D)**, T stage **(E-F)**.


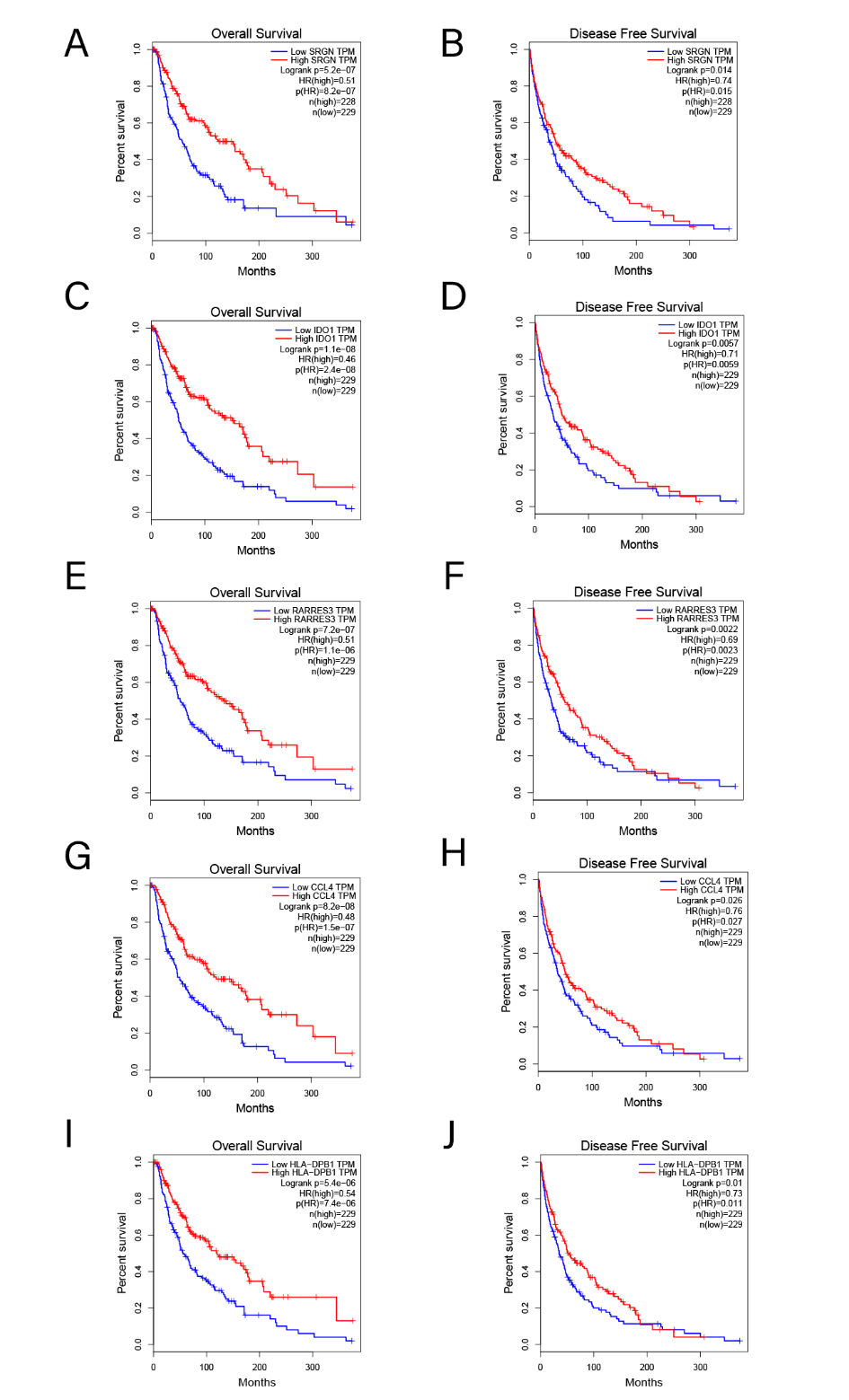


**Supplementary Figure S4. Survival analyses on 5-mRNA signature in the GEPIA database.** Overall survival **(A)** and disease free survival **(B)** analysis related to 5-mRNA expression levels in the GEPIA database.


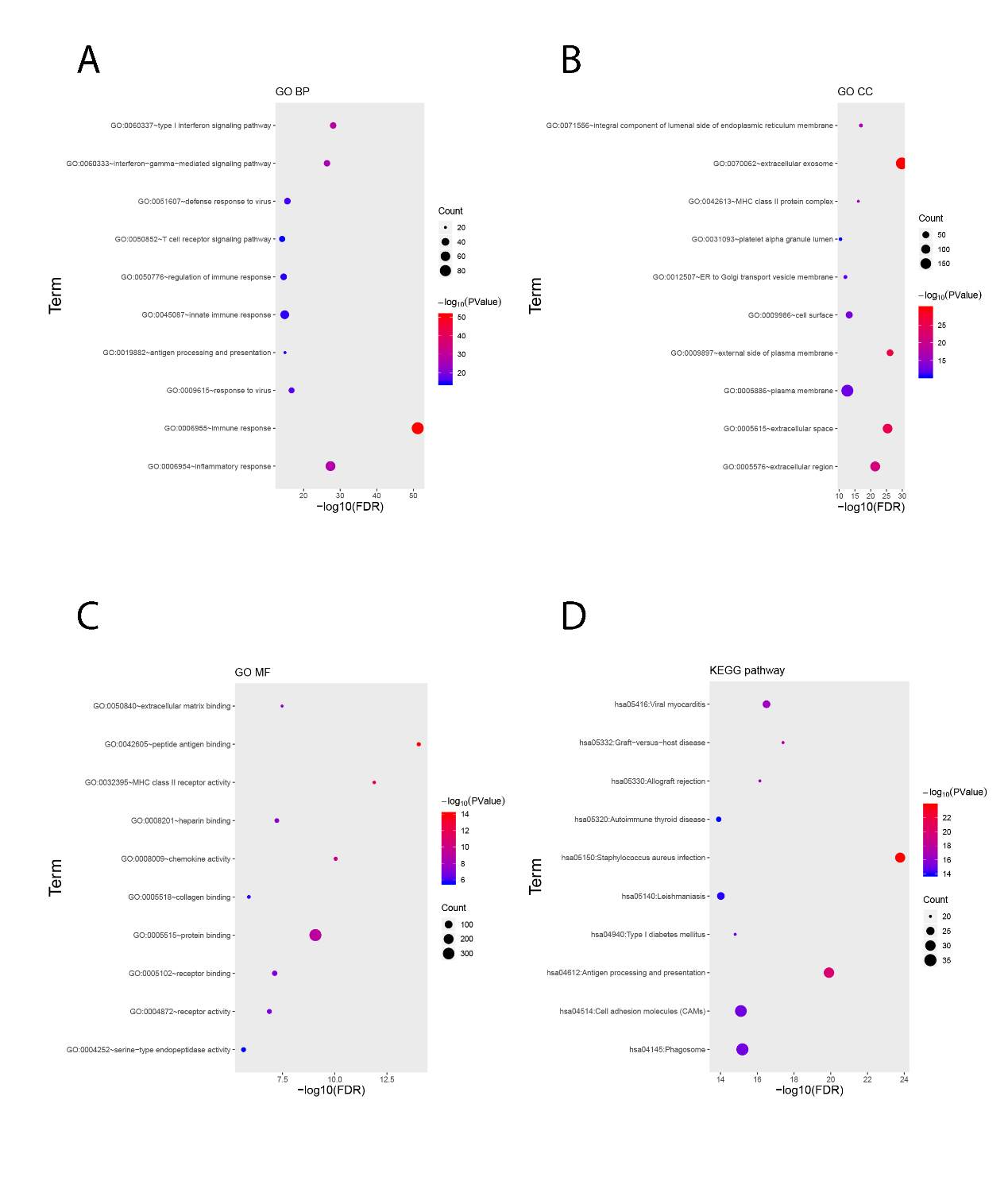


**Supplementary Figure S5.** Functional annotation of all the genes of the blue module to DAVID's website for GO and KEGG analysis.


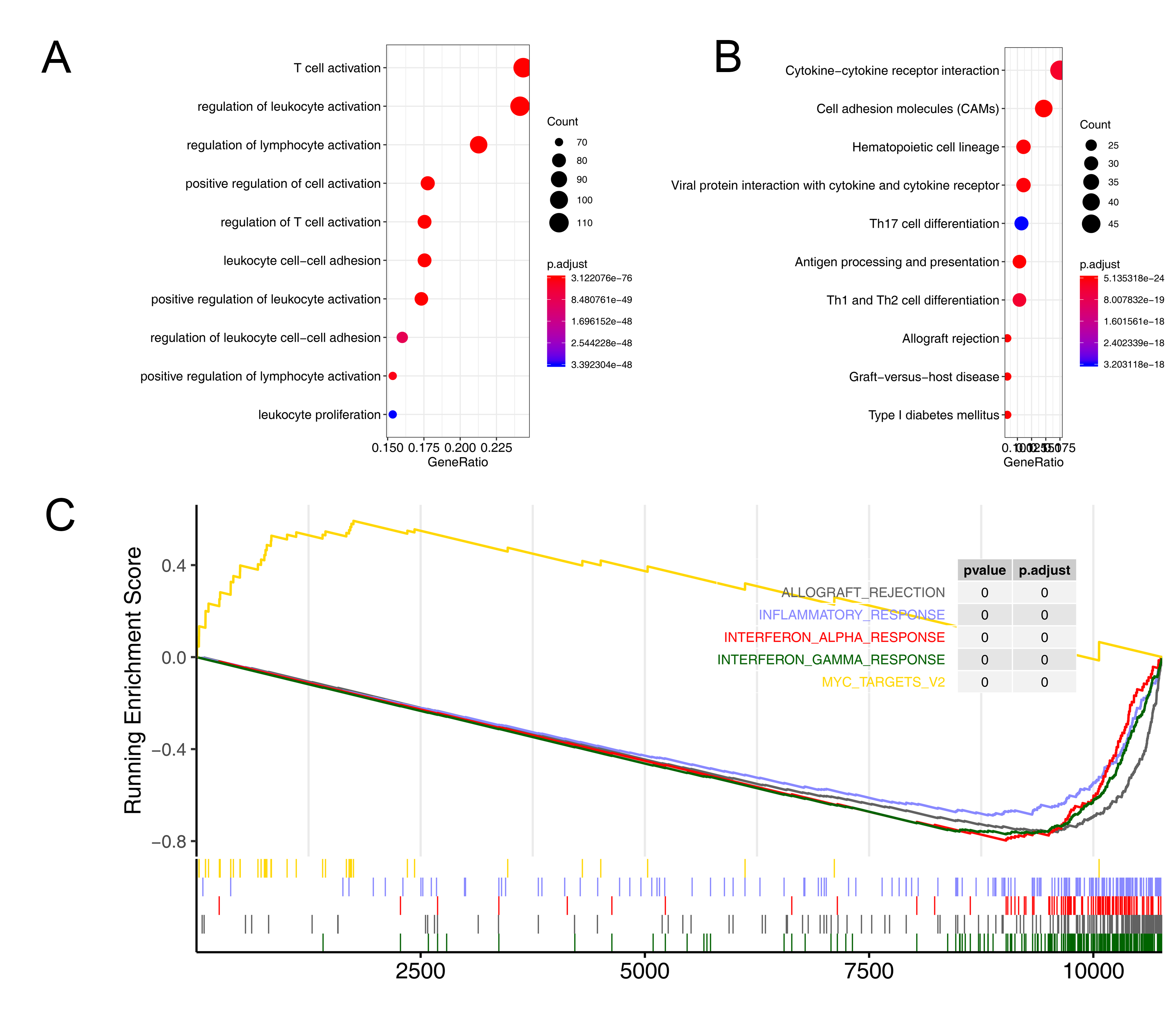


**Supplementary Figure S6.** **(A)** GO, **(B)** KEGG, **and (C)** GSEA analyses were performed for risk score.


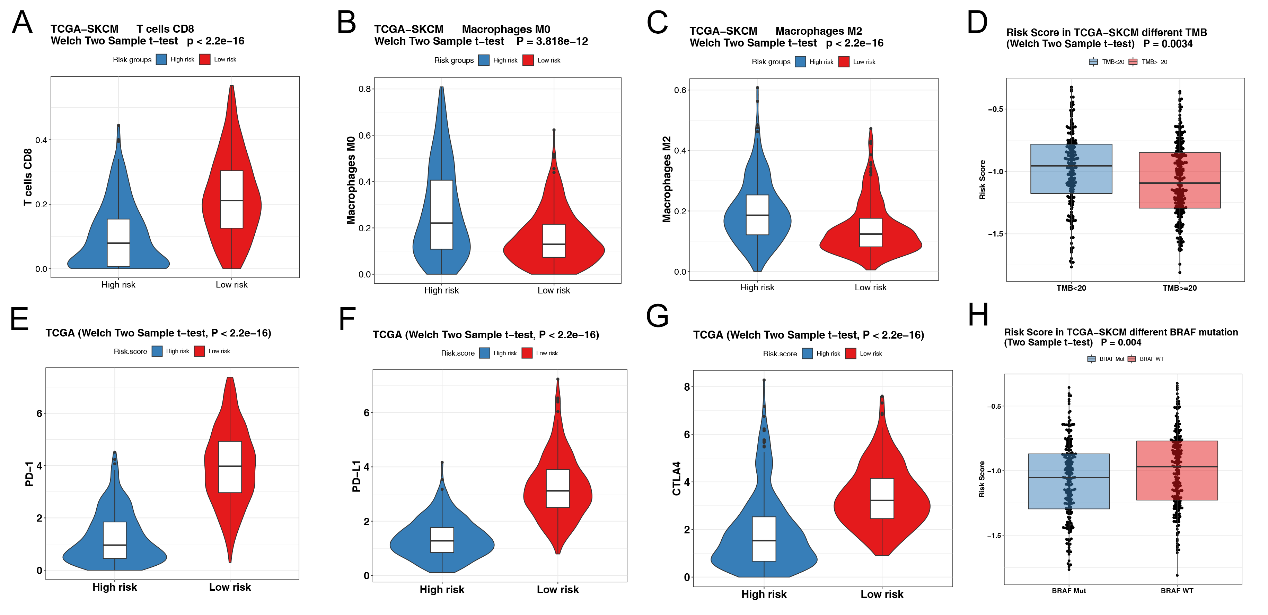


**Supplementary Figure S7.** **(A)** CD8 T cells, **(B)** macrophages M0, **(C)** M2 cells infiltration in high- and low-risk groups. (D) Risk score distribution for different TMB levels. Immune checkpoint **(E)** PD-1, **(F)** PD-L1, **(G)** CTLA4 expression levels in different risk groups. (H) Risk score levels for different BRAF mutation states.

**Supplementary Table S1.** Function annotation results of the blue module.

| Category | Term | P-Value | Fold Enrichment |
| --- | --- | --- | --- |
|  | immune response | ＜0.001 | 7.17 |
|  | type I interferon signaling pathway | ＜0.001 | 15.89 |
|  | inflammatory response | ＜0.001 | 5.45 |
|  | interferon-gamma-mediated signaling pathway | ＜0.001 | 14.32 |
| GO BP | response to virus | ＜0.001 | 8.35 |
|  | defense response to virus | ＜0.001 | 6.36 |
|  | antigen processing and presentation | ＜0.001 | 11.93 |
|  | innate immune response | ＜0.001 | 3.81 |
|  | regulation of immune response | ＜0.001 | 5.90 |
|  | T cell receptor signaling pathway | ＜0.001 | 6.43 |
|  | extracellular exosome | ＜0.001 | 2.29 |
|  | external side of plasma membrane | ＜0.001 | 7.46 |
|  | extracellular space | ＜0.001 | 2.91 |
|  | extracellular region | ＜0.001 | 2.54 |
| GO CC | integral component of lumenal side of endoplasmic reticulum membrane | ＜0.001 | 19.82 |
|  | MHC class II protein complex | ＜0.001 | 23.05 |
|  | cell surface | ＜0.001 | 3.31 |
|  | plasma membrane | ＜0.001 | 1.61 |
|  | ER to Golgi transport vesicle membrane | ＜0.001 | 11.05 |
|  | platelet alpha granule lumen | ＜0.001 | 9.84 |
|  | peptide antigen binding | ＜0.001 | 18.01 |
|  | MHC class II receptor activity | ＜0.001 | 24.66 |
|  | chemokine activity | ＜0.001 | 10.29 |
|  | protein binding | ＜0.001 | 1.26 |
| GO MF | extracellular matrix binding | ＜0.001 | 12.93 |
|  | heparin binding | ＜0.001 | 4.41 |
|  | receptor binding | ＜0.001 | 3.05 |
|  | receptor activity | ＜0.001 | 3.72 |
|  | collagen binding | ＜0.001 | 6.73 |
|  | serine-type endopeptidase activity | ＜0.001 | 3.16 |
|  | Staphylococcus aureus infection | ＜0.001 | 18.01 |
|  | Antigen processing and presentation | ＜0.001 | 24.66 |
|  | Graft-versus-host disease | ＜0.001 | 10.29 |
|  | Viral myocarditis | ＜0.001 | 1.26 |
| KEGG PATHWAY | Allograft rejection | ＜0.001 | 12.93 |
|  | Phagosome | ＜0.001 | 4.41 |
|  | Cell adhesion molecules (CAMs) | ＜0.001 | 3.05 |
|  | Type I diabetes mellitus | ＜0.001 | 3.72 |
|  | Leishmaniasis | ＜0.001 | 6.73 |
|  | Autoimmune thyroid disease | ＜0.001 | 3.16 |

**Supplementary Table S2.** Function annotation results of the prognostic signature.

|  | Description | GeneRatio | pvalue | p.adjust | Count |
| --- | --- | --- | --- | --- | --- |
| GO | T cell activation | 111/456 | 0.00 | 0.00 | 111.00 |
|  | regulation of leukocyte activation | 110/456 | 0.00 | 0.00 | 110.00 |
|  | regulation of lymphocyte activation | 97/456 | 0.00 | 0.00 | 97.00 |
|  | regulation of T cell activation | 80/456 | 0.00 | 0.00 | 80.00 |
|  | positive regulation of cell activation | 81/456 | 0.00 | 0.00 | 81.00 |
|  | positive regulation of leukocyte activation | 79/456 | 0.00 | 0.00 | 79.00 |
|  | leukocyte cell-cell adhesion | 80/456 | 0.00 | 0.00 | 80.00 |
|  | positive regulation of lymphocyte activation | 70/456 | 0.00 | 0.00 | 70.00 |
|  | regulation of leukocyte cell-cell adhesion | 73/456 | 0.00 | 0.00 | 73.00 |
|  | leukocyte proliferation | 70/456 | 0.00 | 0.00 | 70.00 |
|  | mononuclear cell proliferation | 67/456 | 0.00 | 0.00 | 67.00 |
|  | lymphocyte proliferation | 66/456 | 0.00 | 0.00 | 66.00 |
|  | leukocyte differentiation | 87/456 | 0.00 | 0.00 | 87.00 |
|  | regulation of leukocyte proliferation | 62/456 | 0.00 | 0.00 | 62.00 |
|  | positive regulation of leukocyte cell-cell adhesion | 62/456 | 0.00 | 0.00 | 62.00 |
|  | positive regulation of T cell activation | 60/456 | 0.00 | 0.00 | 60.00 |
|  | regulation of mononuclear cell proliferation | 60/456 | 0.00 | 0.00 | 60.00 |
|  | regulation of lymphocyte proliferation | 59/456 | 0.00 | 0.00 | 59.00 |
|  | lymphocyte differentiation | 70/456 | 0.00 | 0.00 | 70.00 |
| KEGG | Cell adhesion molecules (CAMs) | 41/280 | 0.00 | 0.00 | 41.00 |
|  | Allograft rejection | 23/280 | 0.00 | 0.00 | 23.00 |
|  | Graft-versus-host disease | 23/280 | 0.00 | 0.00 | 23.00 |
|  | Antigen processing and presentation | 29/280 | 0.00 | 0.00 | 29.00 |
|  | Type I diabetes mellitus | 23/280 | 0.00 | 0.00 | 23.00 |
|  | Hematopoietic cell lineage | 31/280 | 0.00 | 0.00 | 31.00 |
|  | Viral protein interaction with cytokine and cytokine receptor | 31/280 | 0.00 | 0.00 | 31.00 |
|  | Cytokine-cytokine receptor interaction | 49/280 | 0.00 | 0.00 | 49.00 |
|  | Th1 and Th2 cell differentiation | 29/280 | 0.00 | 0.00 | 29.00 |
|  | Th17 cell differentiation | 30/280 | 0.00 | 0.00 | 30.00 |
|  | Autoimmune thyroid disease | 22/280 | 0.00 | 0.00 | 22.00 |
|  | Viral myocarditis | 23/280 | 0.00 | 0.00 | 23.00 |
|  | Chemokine signaling pathway | 37/280 | 0.00 | 0.00 | 37.00 |
|  | Staphylococcus aureus infection | 27/280 | 0.00 | 0.00 | 27.00 |
|  | Intestinal immune network for IgA production | 20/280 | 0.00 | 0.00 | 20.00 |
|  | Inflammatory bowel disease (IBD) | 22/280 | 0.00 | 0.00 | 22.00 |
|  | Leishmaniasis | 23/280 | 0.00 | 0.00 | 23.00 |
|  | Primary immunodeficiency | 17/280 | 0.00 | 0.00 | 17.00 |
|  | Osteoclast differentiation | 28/280 | 0.00 | 0.00 | 28.00 |

**Supplementary Table S3. Correlation analysis of immune cells and risk score.**

| **Immune cell 1** | **Immune cell 2** | **P value** | **Pearson correlation coefficient** | **Significant** | **Change(The positive and negative correlation)** |
| --- | --- | --- | --- | --- | --- |
| T.cells.CD8 | T.cells.CD4.memory.activated | 2.61E-24 | 0.454398 | NS | NS |
| T.cells.CD4.memory.activated | T.cells.CD8 | 2.61E-24 | 0.454398 | NS | NS |
| Neutrophils | Mast.cells.activated | 7.84E-23 | 0.440945 | NS | NS |
| Mast.cells.activated | Neutrophils | 7.84E-23 | 0.440945 | NS | NS |
| Score | Macrophages.M0 | 9.23E-15 | 0.354375 | NS | NS |
| Macrophages.M0 | Score | 9.23E-15 | 0.354375 | NS | NS |
| Score | Macrophages.M2 | 3.62E-12 | 0.319928 | NS | NS |
| Macrophages.M2 | Score | 3.62E-12 | 0.319928 | NS | NS |
| NK.cells.resting | Mast.cells.activated | 3.99E-12 | 0.31933 | NS | NS |
| Mast.cells.activated | NK.cells.resting | 3.99E-12 | 0.31933 | NS | NS |
| Monocytes | Macrophages.M2 | 2.29E-11 | 0.308351 | NS | NS |
| Macrophages.M2 | Monocytes | 2.29E-11 | 0.308351 | NS | NS |
| T.cells.CD4.memory.resting | Mast.cells.resting | 7.64E-11 | 0.300487 | NS | NS |
| Mast.cells.resting | T.cells.CD4.memory.resting | 7.64E-11 | 0.300487 | NS | NS |
| T.cells.regulatory..Tregs. | B.cells.memory | 9.91E-11 | 0.298756 | NS | NS |
| B.cells.memory | T.cells.regulatory..Tregs. | 9.91E-11 | 0.298756 | NS | NS |
| Score | NK.cells.resting | 1.23E-10 | 0.297305 | NS | NS |
| NK.cells.resting | Score | 1.23E-10 | 0.297305 | NS | NS |
| T.cells.CD4.memory.activated | Macrophages.M1 | 3.08E-10 | 0.291097 | NS | NS |
| Macrophages.M1 | T.cells.CD4.memory.activated | 3.08E-10 | 0.291097 | NS | NS |
| T.cells.CD8 | Macrophages.M1 | 4.64E-10 | 0.28827 | NS | NS |
| Macrophages.M1 | T.cells.CD8 | 4.64E-10 | 0.28827 | NS | NS |
| T.cells.follicular.helper | T.cells.CD8 | 4.87E-10 | 0.287929 | NS | NS |
| T.cells.CD8 | T.cells.follicular.helper | 4.87E-10 | 0.287929 | NS | NS |
| T.cells.CD8 | NK.cells.activated | 4.96E-10 | 0.287811 | NS | NS |
| NK.cells.activated | T.cells.CD8 | 4.96E-10 | 0.287811 | NS | NS |
| T.cells.regulatory..Tregs. | T.cells.CD4.naive | 1.38E-09 | 0.280612 | NS | NS |
| T.cells.CD4.naive | T.cells.regulatory..Tregs. | 1.38E-09 | 0.280612 | NS | NS |
| B.cells.naive | B.cells.memory | 1.36E-08 | 0.26365 | NS | NS |
| B.cells.memory | B.cells.naive | 1.36E-08 | 0.26365 | NS | NS |
| T.cells.CD4.memory.resting | Score | 2.16E-08 | 0.260109 | NS | NS |
| Score | T.cells.CD4.memory.resting | 2.16E-08 | 0.260109 | NS | NS |
| T.cells.regulatory..Tregs. | B.cells.naive | 6.58E-08 | 0.251272 | NS | NS |
| B.cells.naive | T.cells.regulatory..Tregs. | 6.58E-08 | 0.251272 | NS | NS |
| T.cells.CD4.naive | NK.cells.resting | 1.12E-07 | 0.246935 | NS | NS |
| NK.cells.resting | T.cells.CD4.naive | 1.12E-07 | 0.246935 | NS | NS |
| T.cells.CD4.naive | B.cells.memory | 2.50E-07 | 0.240232 | NS | NS |
| B.cells.memory | T.cells.CD4.naive | 2.50E-07 | 0.240232 | NS | NS |
| Mast.cells.activated | Dendritic.cells.activated | 3.96E-07 | 0.2363 | NS | NS |
| Dendritic.cells.activated | Mast.cells.activated | 3.96E-07 | 0.2363 | NS | NS |
| NK.cells.activated | Mast.cells.resting | 1.21E-06 | 0.226468 | NS | NS |
| Mast.cells.resting | NK.cells.activated | 1.21E-06 | 0.226468 | NS | NS |
| T.cells.CD4.memory.resting | Macrophages.M2 | 1.39E-06 | 0.225198 | NS | NS |
| Macrophages.M2 | T.cells.CD4.memory.resting | 1.39E-06 | 0.225198 | NS | NS |
| Score | Mast.cells.resting | 1.54E-06 | 0.224265 | NS | NS |
| Mast.cells.resting | Score | 1.54E-06 | 0.224265 | NS | NS |
| NK.cells.resting | Macrophages.M0 | 3.69E-06 | 0.21619 | NS | NS |
| Macrophages.M0 | NK.cells.resting | 3.69E-06 | 0.21619 | NS | NS |
| Neutrophils | Dendritic.cells.activated | 6.91E-06 | 0.210172 | NS | NS |
| Dendritic.cells.activated | Neutrophils | 6.91E-06 | 0.210172 | NS | NS |
| Mast.cells.activated | Macrophages.M0 | 1.26E-05 | 0.20427 | NS | NS |
| Macrophages.M0 | Mast.cells.activated | 1.26E-05 | 0.20427 | NS | NS |
| Mast.cells.resting | Macrophages.M2 | 5.85E-05 | 0.18827 | NS | NS |
| Macrophages.M2 | Mast.cells.resting | 5.85E-05 | 0.18827 | NS | NS |
| Mast.cells.resting | Dendritic.cells.resting | 9.66E-05 | 0.182761 | NS | NS |
| Dendritic.cells.resting | Mast.cells.resting | 9.66E-05 | 0.182761 | NS | NS |
| T.cells.CD4.memory.resting | Monocytes | 0.0001 | 0.182327 | Sig | Positive |
| Monocytes | T.cells.CD4.memory.resting | 0.0001 | 0.182327 | Sig | Positive |
| T.cells.CD4.naive | B.cells.naive | 0.000121 | 0.180255 | Sig | Positive |
| B.cells.naive | T.cells.CD4.naive | 0.000121 | 0.180255 | Sig | Positive |
| NK.cells.resting | Eosinophils | 0.000139 | 0.178653 | Sig | Positive |
| Eosinophils | NK.cells.resting | 0.000139 | 0.178653 | Sig | Positive |
| T.cells.follicular.helper | Macrophages.M1 | 0.000149 | 0.177876 | Sig | Positive |
| Macrophages.M1 | T.cells.follicular.helper | 0.000149 | 0.177876 | Sig | Positive |
| T.cells.gamma.delta | Plasma.cells | 0.00017 | 0.176345 | Sig | Positive |
| Plasma.cells | T.cells.gamma.delta | 0.00017 | 0.176345 | Sig | Positive |
| NK.cells.activated | Macrophages.M1 | 0.000185 | 0.175388 | Sig | Positive |
| Macrophages.M1 | NK.cells.activated | 0.000185 | 0.175388 | Sig | Positive |
| Monocytes | Mast.cells.resting | 0.000369 | 0.167167 | Sig | Positive |
| Mast.cells.resting | Monocytes | 0.000369 | 0.167167 | Sig | Positive |
| T.cells.gamma.delta | Macrophages.M1 | 0.00099 | 0.154743 | Sig | Positive |
| Macrophages.M1 | T.cells.gamma.delta | 0.00099 | 0.154743 | Sig | Positive |
| T.cells.gamma.delta | T.cells.CD4.memory.activated | 0.001303 | 0.151123 | Sig | Positive |
| T.cells.CD4.memory.activated | T.cells.gamma.delta | 0.001303 | 0.151123 | Sig | Positive |
| Macrophages.M0 | Eosinophils | 0.002004 | 0.145278 | Sig | Positive |
| Eosinophils | Macrophages.M0 | 0.002004 | 0.145278 | Sig | Positive |
| Score | Dendritic.cells.activated | 0.002444 | 0.14251 | Sig | Positive |
| Dendritic.cells.activated | Score | 0.002444 | 0.14251 | Sig | Positive |
| Score | Eosinophils | 0.003286 | 0.138295 | Sig | Positive |
| Eosinophils | Score | 0.003286 | 0.138295 | Sig | Positive |
| Macrophages.M2 | Dendritic.cells.resting | 0.003286 | 0.138294 | Sig | Positive |
| Dendritic.cells.resting | Macrophages.M2 | 0.003286 | 0.138294 | Sig | Positive |
| NK.cells.activated | Dendritic.cells.resting | 0.003982 | 0.135496 | Sig | Positive |
| Dendritic.cells.resting | NK.cells.activated | 0.003982 | 0.135496 | Sig | Positive |
| T.cells.CD4.memory.resting | NK.cells.resting | 0.004235 | 0.134587 | Sig | Positive |
| NK.cells.resting | T.cells.CD4.memory.resting | 0.004235 | 0.134587 | Sig | Positive |
| T.cells.CD4.naive | T.cells.CD4.memory.resting | 0.00446 | 0.133821 | Sig | Positive |
| T.cells.CD4.memory.resting | T.cells.CD4.naive | 0.00446 | 0.133821 | Sig | Positive |
| Monocytes | Dendritic.cells.resting | 0.004704 | 0.133027 | Sig | Positive |
| Dendritic.cells.resting | Monocytes | 0.004704 | 0.133027 | Sig | Positive |
| Score | Mast.cells.activated | 0.006431 | 0.128282 | Sig | Positive |
| Mast.cells.activated | Score | 0.006431 | 0.128282 | Sig | Positive |
| T.cells.gamma.delta | T.cells.CD8 | 0.007124 | 0.126696 | Sig | Positive |
| T.cells.CD8 | T.cells.gamma.delta | 0.007124 | 0.126696 | Sig | Positive |
| Mast.cells.resting | Eosinophils | 0.014326 | 0.115381 | Sig | Positive |
| Eosinophils | Mast.cells.resting | 0.014326 | 0.115381 | Sig | Positive |
| Macrophages.M1 | Dendritic.cells.resting | 0.016632 | 0.112842 | Sig | Positive |
| Dendritic.cells.resting | Macrophages.M1 | 0.016632 | 0.112842 | Sig | Positive |
| T.cells.CD4.naive | Score | 0.018852 | 0.110674 | Sig | Positive |
| Score | T.cells.CD4.naive | 0.018852 | 0.110674 | Sig | Positive |
| T.cells.CD4.memory.resting | Eosinophils | 0.023449 | 0.106814 | Sig | Positive |
| Eosinophils | T.cells.CD4.memory.resting | 0.023449 | 0.106814 | Sig | Positive |
| Score | B.cells.naive | 0.023452 | 0.106811 | Sig | Positive |
| B.cells.naive | Score | 0.023452 | 0.106811 | Sig | Positive |
| T.cells.regulatory..Tregs. | T.cells.CD8 | 0.025292 | 0.105448 | Sig | Positive |
| T.cells.CD8 | T.cells.regulatory..Tregs. | 0.025292 | 0.105448 | Sig | Positive |
| T.cells.CD8 | Plasma.cells | 0.044764 | 0.094655 | Sig | Positive |
| Plasma.cells | T.cells.CD8 | 0.044764 | 0.094655 | Sig | Positive |
| T.cells.follicular.helper | NK.cells.activated | 0.05665 | 0.089918 | NS | NS |
| NK.cells.activated | T.cells.follicular.helper | 0.05665 | 0.089918 | NS | NS |
| T.cells.CD4.memory.activated | Plasma.cells | 0.095296 | 0.078732 | NS | NS |
| Plasma.cells | T.cells.CD4.memory.activated | 0.095296 | 0.078732 | NS | NS |
| T.cells.CD4.memory.resting | B.cells.naive | 0.098223 | 0.078046 | NS | NS |
| B.cells.naive | T.cells.CD4.memory.resting | 0.098223 | 0.078046 | NS | NS |
| Plasma.cells | Macrophages.M1 | 0.126968 | 0.072051 | NS | NS |
| Macrophages.M1 | Plasma.cells | 0.126968 | 0.072051 | NS | NS |
| Neutrophils | Dendritic.cells.resting | 0.140105 | 0.06966 | NS | NS |
| Dendritic.cells.resting | Neutrophils | 0.140105 | 0.06966 | NS | NS |
| Score | Neutrophils | 0.14201 | 0.069328 | NS | NS |
| Neutrophils | Score | 0.14201 | 0.069328 | NS | NS |
| Score | Dendritic.cells.resting | 0.157013 | 0.066824 | NS | NS |
| Dendritic.cells.resting | Score | 0.157013 | 0.066824 | NS | NS |
| NK.cells.resting | Neutrophils | 0.159331 | 0.066453 | NS | NS |
| Neutrophils | NK.cells.resting | 0.159331 | 0.066453 | NS | NS |
| Mast.cells.activated | Eosinophils | 0.165167 | 0.065538 | NS | NS |
| Eosinophils | Mast.cells.activated | 0.165167 | 0.065538 | NS | NS |
| T.cells.follicular.helper | B.cells.naive | 0.178333 | 0.063559 | NS | NS |
| B.cells.naive | T.cells.follicular.helper | 0.178333 | 0.063559 | NS | NS |
| T.cells.gamma.delta | NK.cells.activated | 0.203531 | 0.060055 | NS | NS |
| NK.cells.activated | T.cells.gamma.delta | 0.203531 | 0.060055 | NS | NS |
| Plasma.cells | NK.cells.activated | 0.219826 | 0.057953 | NS | NS |
| NK.cells.activated | Plasma.cells | 0.219826 | 0.057953 | NS | NS |
| T.cells.CD4.memory.resting | Dendritic.cells.activated | 0.236315 | 0.055938 | NS | NS |
| Dendritic.cells.activated | T.cells.CD4.memory.resting | 0.236315 | 0.055938 | NS | NS |
| T.cells.CD4.naive | Mast.cells.activated | 0.236532 | 0.055912 | NS | NS |
| Mast.cells.activated | T.cells.CD4.naive | 0.236532 | 0.055912 | NS | NS |
| Plasma.cells | B.cells.naive | 0.258886 | 0.053333 | NS | NS |
| B.cells.naive | Plasma.cells | 0.258886 | 0.053333 | NS | NS |
| T.cells.gamma.delta | Eosinophils | 0.311963 | 0.04777 | NS | NS |
| Eosinophils | T.cells.gamma.delta | 0.311963 | 0.04777 | NS | NS |
| T.cells.follicular.helper | Plasma.cells | 0.345442 | 0.044577 | NS | NS |
| Plasma.cells | T.cells.follicular.helper | 0.345442 | 0.044577 | NS | NS |
| T.cells.regulatory..Tregs. | Plasma.cells | 0.400608 | 0.039718 | NS | NS |
| Plasma.cells | T.cells.regulatory..Tregs. | 0.400608 | 0.039718 | NS | NS |
| T.cells.follicular.helper | Dendritic.cells.activated | 0.427032 | 0.037534 | NS | NS |
| Dendritic.cells.activated | T.cells.follicular.helper | 0.427032 | 0.037534 | NS | NS |
| NK.cells.resting | Macrophages.M2 | 0.444935 | 0.036099 | NS | NS |
| Macrophages.M2 | NK.cells.resting | 0.444935 | 0.036099 | NS | NS |
| Macrophages.M0 | Dendritic.cells.activated | 0.445811 | 0.036029 | NS | NS |
| Dendritic.cells.activated | Macrophages.M0 | 0.445811 | 0.036029 | NS | NS |
| T.cells.CD4.naive | Monocytes | 0.452695 | 0.035487 | NS | NS |
| Monocytes | T.cells.CD4.naive | 0.452695 | 0.035487 | NS | NS |
| Mast.cells.resting | Macrophages.M1 | 0.488425 | 0.032742 | NS | NS |
| Macrophages.M1 | Mast.cells.resting | 0.488425 | 0.032742 | NS | NS |
| T.cells.regulatory..Tregs. | NK.cells.activated | 0.495122 | 0.03224 | NS | NS |
| NK.cells.activated | T.cells.regulatory..Tregs. | 0.495122 | 0.03224 | NS | NS |
| T.cells.CD4.memory.activated | Neutrophils | 0.495189 | 0.032235 | NS | NS |
| Neutrophils | T.cells.CD4.memory.activated | 0.495189 | 0.032235 | NS | NS |
| Macrophages.M2 | Dendritic.cells.activated | 0.564309 | 0.027245 | NS | NS |
| Dendritic.cells.activated | Macrophages.M2 | 0.564309 | 0.027245 | NS | NS |
| T.cells.CD4.memory.activated | B.cells.memory | 0.57792 | 0.026299 | NS | NS |
| B.cells.memory | T.cells.CD4.memory.activated | 0.57792 | 0.026299 | NS | NS |
| NK.cells.resting | Dendritic.cells.activated | 0.585285 | 0.025791 | NS | NS |
| Dendritic.cells.activated | NK.cells.resting | 0.585285 | 0.025791 | NS | NS |
| T.cells.follicular.helper | Score | 0.630001 | 0.022769 | NS | NS |
| Score | T.cells.follicular.helper | 0.630001 | 0.022769 | NS | NS |
| Dendritic.cells.resting | Dendritic.cells.activated | 0.631181 | 0.022691 | NS | NS |
| Dendritic.cells.activated | Dendritic.cells.resting | 0.631181 | 0.022691 | NS | NS |
| T.cells.follicular.helper | Dendritic.cells.resting | 0.72137 | 0.016857 | NS | NS |
| Dendritic.cells.resting | T.cells.follicular.helper | 0.72137 | 0.016857 | NS | NS |
| T.cells.gamma.delta | B.cells.memory | 0.738441 | 0.015784 | NS | NS |
| B.cells.memory | T.cells.gamma.delta | 0.738441 | 0.015784 | NS | NS |
| Macrophages.M2 | Eosinophils | 0.791183 | 0.012516 | NS | NS |
| Eosinophils | Macrophages.M2 | 0.791183 | 0.012516 | NS | NS |
| T.cells.CD4.naive | Dendritic.cells.activated | 0.802016 | 0.011852 | NS | NS |
| Dendritic.cells.activated | T.cells.CD4.naive | 0.802016 | 0.011852 | NS | NS |
| Dendritic.cells.activated | B.cells.naive | 0.813986 | 0.011122 | NS | NS |
| B.cells.naive | Dendritic.cells.activated | 0.813986 | 0.011122 | NS | NS |
| T.cells.CD4.memory.resting | Dendritic.cells.resting | 0.842745 | 0.009378 | NS | NS |
| Dendritic.cells.resting | T.cells.CD4.memory.resting | 0.842745 | 0.009378 | NS | NS |
| NK.cells.resting | Mast.cells.resting | 0.852116 | 0.008812 | NS | NS |
| Mast.cells.resting | NK.cells.resting | 0.852116 | 0.008812 | NS | NS |
| Monocytes | Dendritic.cells.activated | 0.860886 | 0.008284 | NS | NS |
| Dendritic.cells.activated | Monocytes | 0.860886 | 0.008284 | NS | NS |
| NK.cells.activated | Monocytes | 0.86565 | 0.007998 | NS | NS |
| Monocytes | NK.cells.activated | 0.86565 | 0.007998 | NS | NS |
| NK.cells.resting | Monocytes | 0.87912 | 0.007189 | NS | NS |
| Monocytes | NK.cells.resting | 0.87912 | 0.007189 | NS | NS |
| Score | Monocytes | 0.909363 | 0.005381 | NS | NS |
| Monocytes | Score | 0.909363 | 0.005381 | NS | NS |
| Neutrophils | Macrophages.M2 | 0.920689 | 0.004707 | NS | NS |
| Macrophages.M2 | Neutrophils | 0.920689 | 0.004707 | NS | NS |
| T.cells.follicular.helper | Neutrophils | 0.930301 | 0.004135 | NS | NS |
| Neutrophils | T.cells.follicular.helper | 0.930301 | 0.004135 | NS | NS |
| Plasma.cells | Dendritic.cells.activated | 0.94304 | 0.003378 | NS | NS |
| Dendritic.cells.activated | Plasma.cells | 0.94304 | 0.003378 | NS | NS |
| Macrophages.M1 | B.cells.memory | 0.968118 | 0.001889 | NS | NS |
| B.cells.memory | Macrophages.M1 | 0.968118 | 0.001889 | NS | NS |
| T.cells.CD4.memory.resting | Neutrophils | 0.978149 | 0.001295 | NS | NS |
| Neutrophils | T.cells.CD4.memory.resting | 0.978149 | 0.001295 | NS | NS |
| T.cells.follicular.helper | Eosinophils | 0.98222 | 0.001053 | NS | NS |
| Eosinophils | T.cells.follicular.helper | 0.98222 | 0.001053 | NS | NS |
| Eosinophils | B.cells.memory | 0.99747 | -0.00015 | NS | NS |
| B.cells.memory | Eosinophils | 0.99747 | -0.00015 | NS | NS |
| Mast.cells.resting | Dendritic.cells.activated | 0.987265 | -0.00075 | NS | NS |
| Dendritic.cells.activated | Mast.cells.resting | 0.987265 | -0.00075 | NS | NS |
| Monocytes | Eosinophils | 0.976213 | -0.00141 | NS | NS |
| Eosinophils | Monocytes | 0.976213 | -0.00141 | NS | NS |
| T.cells.CD4.memory.activated | Dendritic.cells.resting | 0.946848 | -0.00315 | NS | NS |
| Dendritic.cells.resting | T.cells.CD4.memory.activated | 0.946848 | -0.00315 | NS | NS |
| T.cells.CD4.memory.resting | B.cells.memory | 0.919175 | -0.0048 | NS | NS |
| B.cells.memory | T.cells.CD4.memory.resting | 0.919175 | -0.0048 | NS | NS |
| Plasma.cells | B.cells.memory | 0.903037 | -0.00576 | NS | NS |
| B.cells.memory | Plasma.cells | 0.903037 | -0.00576 | NS | NS |
| T.cells.gamma.delta | Neutrophils | 0.857968 | -0.00846 | NS | NS |
| Neutrophils | T.cells.gamma.delta | 0.857968 | -0.00846 | NS | NS |
| T.cells.CD4.memory.resting | Mast.cells.activated | 0.850394 | -0.00892 | NS | NS |
| Mast.cells.activated | T.cells.CD4.memory.resting | 0.850394 | -0.00892 | NS | NS |
| Eosinophils | Dendritic.cells.activated | 0.834157 | -0.0099 | NS | NS |
| Dendritic.cells.activated | Eosinophils | 0.834157 | -0.0099 | NS | NS |
| Neutrophils | Monocytes | 0.79912 | -0.01203 | NS | NS |
| Monocytes | Neutrophils | 0.79912 | -0.01203 | NS | NS |
| T.cells.gamma.delta | Mast.cells.activated | 0.795184 | -0.01227 | NS | NS |
| Mast.cells.activated | T.cells.gamma.delta | 0.795184 | -0.01227 | NS | NS |
| T.cells.follicular.helper | T.cells.CD4.memory.activated | 0.745633 | -0.01533 | NS | NS |
| T.cells.CD4.memory.activated | T.cells.follicular.helper | 0.745633 | -0.01533 | NS | NS |
| Mast.cells.activated | Dendritic.cells.resting | 0.735315 | -0.01598 | NS | NS |
| Dendritic.cells.resting | Mast.cells.activated | 0.735315 | -0.01598 | NS | NS |
| T.cells.CD4.naive | Eosinophils | 0.733503 | -0.01609 | NS | NS |
| Eosinophils | T.cells.CD4.naive | 0.733503 | -0.01609 | NS | NS |
| T.cells.regulatory..Tregs. | Dendritic.cells.activated | 0.733107 | -0.01612 | NS | NS |
| Dendritic.cells.activated | T.cells.regulatory..Tregs. | 0.733107 | -0.01612 | NS | NS |
| T.cells.CD4.naive | Neutrophils | 0.725706 | -0.01658 | NS | NS |
| Neutrophils | T.cells.CD4.naive | 0.725706 | -0.01658 | NS | NS |
| T.cells.regulatory..Tregs. | Score | 0.721665 | -0.01684 | NS | NS |
| Score | T.cells.regulatory..Tregs. | 0.721665 | -0.01684 | NS | NS |
| Neutrophils | B.cells.naive | 0.638909 | -0.02218 | NS | NS |
| B.cells.naive | Neutrophils | 0.638909 | -0.02218 | NS | NS |
| Neutrophils | Macrophages.M0 | 0.61208 | -0.02397 | NS | NS |
| Macrophages.M0 | Neutrophils | 0.61208 | -0.02397 | NS | NS |
| T.cells.CD4.naive | Macrophages.M0 | 0.540445 | -0.02893 | NS | NS |
| Macrophages.M0 | T.cells.CD4.naive | 0.540445 | -0.02893 | NS | NS |
| T.cells.CD4.naive | Mast.cells.resting | 0.535816 | -0.02926 | NS | NS |
| Mast.cells.resting | T.cells.CD4.naive | 0.535816 | -0.02926 | NS | NS |
| Plasma.cells | Neutrophils | 0.520991 | -0.03033 | NS | NS |
| Neutrophils | Plasma.cells | 0.520991 | -0.03033 | NS | NS |
| T.cells.gamma.delta | T.cells.follicular.helper | 0.515313 | -0.03075 | NS | NS |
| T.cells.follicular.helper | T.cells.gamma.delta | 0.515313 | -0.03075 | NS | NS |
| T.cells.regulatory..Tregs. | Mast.cells.activated | 0.503481 | -0.03162 | NS | NS |
| Mast.cells.activated | T.cells.regulatory..Tregs. | 0.503481 | -0.03162 | NS | NS |
| T.cells.CD4.memory.activated | NK.cells.activated | 0.493696 | -0.03235 | NS | NS |
| NK.cells.activated | T.cells.CD4.memory.activated | 0.493696 | -0.03235 | NS | NS |
| Plasma.cells | Dendritic.cells.resting | 0.48026 | -0.03336 | NS | NS |
| Dendritic.cells.resting | Plasma.cells | 0.48026 | -0.03336 | NS | NS |
| T.cells.regulatory..Tregs. | Dendritic.cells.resting | 0.445531 | -0.03605 | NS | NS |
| Dendritic.cells.resting | T.cells.regulatory..Tregs. | 0.445531 | -0.03605 | NS | NS |
| T.cells.CD4.memory.activated | Dendritic.cells.activated | 0.418155 | -0.03826 | NS | NS |
| Dendritic.cells.activated | T.cells.CD4.memory.activated | 0.418155 | -0.03826 | NS | NS |
| Neutrophils | Mast.cells.resting | 0.411913 | -0.03877 | NS | NS |
| Mast.cells.resting | Neutrophils | 0.411913 | -0.03877 | NS | NS |
| Neutrophils | Eosinophils | 0.385086 | -0.04104 | NS | NS |
| Eosinophils | Neutrophils | 0.385086 | -0.04104 | NS | NS |
| NK.cells.activated | B.cells.memory | 0.365673 | -0.04274 | NS | NS |
| B.cells.memory | NK.cells.activated | 0.365673 | -0.04274 | NS | NS |
| T.cells.CD8 | Dendritic.cells.resting | 0.362552 | -0.04302 | NS | NS |
| Dendritic.cells.resting | T.cells.CD8 | 0.362552 | -0.04302 | NS | NS |
| Neutrophils | B.cells.memory | 0.344057 | -0.04471 | NS | NS |
| B.cells.memory | Neutrophils | 0.344057 | -0.04471 | NS | NS |
| T.cells.CD8 | B.cells.memory | 0.331326 | -0.0459 | NS | NS |
| B.cells.memory | T.cells.CD8 | 0.331326 | -0.0459 | NS | NS |
| T.cells.gamma.delta | Dendritic.cells.activated | 0.292557 | -0.04972 | NS | NS |
| Dendritic.cells.activated | T.cells.gamma.delta | 0.292557 | -0.04972 | NS | NS |
| Dendritic.cells.activated | B.cells.memory | 0.286228 | -0.05038 | NS | NS |
| B.cells.memory | Dendritic.cells.activated | 0.286228 | -0.05038 | NS | NS |
| NK.cells.resting | B.cells.memory | 0.270805 | -0.05202 | NS | NS |
| B.cells.memory | NK.cells.resting | 0.270805 | -0.05202 | NS | NS |
| T.cells.follicular.helper | Macrophages.M2 | 0.267111 | -0.05242 | NS | NS |
| Macrophages.M2 | T.cells.follicular.helper | 0.267111 | -0.05242 | NS | NS |
| Dendritic.cells.resting | B.cells.memory | 0.26215 | -0.05297 | NS | NS |
| B.cells.memory | Dendritic.cells.resting | 0.26215 | -0.05297 | NS | NS |
| Plasma.cells | Eosinophils | 0.258114 | -0.05342 | NS | NS |
| Eosinophils | Plasma.cells | 0.258114 | -0.05342 | NS | NS |
| Mast.cells.activated | B.cells.memory | 0.25733 | -0.05351 | NS | NS |
| B.cells.memory | Mast.cells.activated | 0.25733 | -0.05351 | NS | NS |
| T.cells.follicular.helper | Monocytes | 0.241418 | -0.05533 | NS | NS |
| Monocytes | T.cells.follicular.helper | 0.241418 | -0.05533 | NS | NS |
| T.cells.follicular.helper | B.cells.memory | 0.232085 | -0.05645 | NS | NS |
| B.cells.memory | T.cells.follicular.helper | 0.232085 | -0.05645 | NS | NS |
| NK.cells.activated | Dendritic.cells.activated | 0.230507 | -0.05664 | NS | NS |
| Dendritic.cells.activated | NK.cells.activated | 0.230507 | -0.05664 | NS | NS |
| T.cells.CD4.naive | T.cells.CD4.memory.activated | 0.229776 | -0.05672 | NS | NS |
| T.cells.CD4.memory.activated | T.cells.CD4.naive | 0.229776 | -0.05672 | NS | NS |
| T.cells.CD4.memory.resting | Macrophages.M0 | 0.223024 | -0.05755 | NS | NS |
| Macrophages.M0 | T.cells.CD4.memory.resting | 0.223024 | -0.05755 | NS | NS |
| T.cells.regulatory..Tregs. | T.cells.follicular.helper | 0.21972 | -0.05797 | NS | NS |
| T.cells.follicular.helper | T.cells.regulatory..Tregs. | 0.21972 | -0.05797 | NS | NS |
| Plasma.cells | Mast.cells.activated | 0.209634 | -0.05925 | NS | NS |
| Mast.cells.activated | Plasma.cells | 0.209634 | -0.05925 | NS | NS |
| Dendritic.cells.resting | B.cells.naive | 0.207594 | -0.05952 | NS | NS |
| B.cells.naive | Dendritic.cells.resting | 0.207594 | -0.05952 | NS | NS |
| NK.cells.activated | Neutrophils | 0.206286 | -0.05969 | NS | NS |
| Neutrophils | NK.cells.activated | 0.206286 | -0.05969 | NS | NS |
| Mast.cells.resting | B.cells.memory | 0.205516 | -0.05979 | NS | NS |
| B.cells.memory | Mast.cells.resting | 0.205516 | -0.05979 | NS | NS |
| Monocytes | B.cells.memory | 0.196804 | -0.06096 | NS | NS |
| B.cells.memory | Monocytes | 0.196804 | -0.06096 | NS | NS |
| T.cells.regulatory..Tregs. | Monocytes | 0.193655 | -0.06139 | NS | NS |
| Monocytes | T.cells.regulatory..Tregs. | 0.193655 | -0.06139 | NS | NS |
| T.cells.CD4.naive | Plasma.cells | 0.189832 | -0.06192 | NS | NS |
| Plasma.cells | T.cells.CD4.naive | 0.189832 | -0.06192 | NS | NS |
| Mast.cells.activated | B.cells.naive | 0.173453 | -0.06428 | NS | NS |
| B.cells.naive | Mast.cells.activated | 0.173453 | -0.06428 | NS | NS |
| Monocytes | Macrophages.M1 | 0.17284 | -0.06437 | NS | NS |
| Macrophages.M1 | Monocytes | 0.17284 | -0.06437 | NS | NS |
| Eosinophils | Dendritic.cells.resting | 0.168535 | -0.06502 | NS | NS |
| Dendritic.cells.resting | Eosinophils | 0.168535 | -0.06502 | NS | NS |
| Neutrophils | Macrophages.M1 | 0.160615 | -0.06625 | NS | NS |
| Macrophages.M1 | Neutrophils | 0.160615 | -0.06625 | NS | NS |
| T.cells.regulatory..Tregs. | T.cells.CD4.memory.resting | 0.142111 | -0.06931 | NS | NS |
| T.cells.CD4.memory.resting | T.cells.regulatory..Tregs. | 0.142111 | -0.06931 | NS | NS |
| T.cells.follicular.helper | Mast.cells.resting | 0.123178 | -0.07278 | NS | NS |
| Mast.cells.resting | T.cells.follicular.helper | 0.123178 | -0.07278 | NS | NS |
| T.cells.CD8 | Neutrophils | 0.115777 | -0.07424 | NS | NS |
| Neutrophils | T.cells.CD8 | 0.115777 | -0.07424 | NS | NS |
| Monocytes | Mast.cells.activated | 0.114393 | -0.07453 | NS | NS |
| Mast.cells.activated | Monocytes | 0.114393 | -0.07453 | NS | NS |
| T.cells.CD4.memory.resting | NK.cells.activated | 0.11193 | -0.07504 | NS | NS |
| NK.cells.activated | T.cells.CD4.memory.resting | 0.11193 | -0.07504 | NS | NS |
| NK.cells.activated | B.cells.naive | 0.096201 | -0.07852 | NS | NS |
| B.cells.naive | NK.cells.activated | 0.096201 | -0.07852 | NS | NS |
| T.cells.CD4.naive | Dendritic.cells.resting | 0.093567 | -0.07914 | NS | NS |
| Dendritic.cells.resting | T.cells.CD4.naive | 0.093567 | -0.07914 | NS | NS |
| T.cells.follicular.helper | NK.cells.resting | 0.087392 | -0.08067 | NS | NS |
| NK.cells.resting | T.cells.follicular.helper | 0.087392 | -0.08067 | NS | NS |
| Eosinophils | B.cells.naive | 0.084053 | -0.08153 | NS | NS |
| B.cells.naive | Eosinophils | 0.084053 | -0.08153 | NS | NS |
| Macrophages.M2 | B.cells.naive | 0.076911 | -0.08347 | NS | NS |
| B.cells.naive | Macrophages.M2 | 0.076911 | -0.08347 | NS | NS |
| Mast.cells.activated | Macrophages.M2 | 0.072889 | -0.08463 | NS | NS |
| Macrophages.M2 | Mast.cells.activated | 0.072889 | -0.08463 | NS | NS |
| T.cells.regulatory..Tregs. | Eosinophils | 0.065199 | -0.087 | NS | NS |
| Eosinophils | T.cells.regulatory..Tregs. | 0.065199 | -0.087 | NS | NS |
| NK.cells.resting | Dendritic.cells.resting | 0.064618 | -0.08719 | NS | NS |
| Dendritic.cells.resting | NK.cells.resting | 0.064618 | -0.08719 | NS | NS |
| T.cells.gamma.delta | T.cells.CD4.naive | 0.064287 | -0.08729 | NS | NS |
| T.cells.CD4.naive | T.cells.gamma.delta | 0.064287 | -0.08729 | NS | NS |
| T.cells.regulatory..Tregs. | NK.cells.resting | 0.059316 | -0.08897 | NS | NS |
| NK.cells.resting | T.cells.regulatory..Tregs. | 0.059316 | -0.08897 | NS | NS |
| T.cells.gamma.delta | T.cells.CD4.memory.resting | 0.056811 | -0.08986 | NS | NS |
| T.cells.CD4.memory.resting | T.cells.gamma.delta | 0.056811 | -0.08986 | NS | NS |
| T.cells.regulatory..Tregs. | Macrophages.M1 | 0.056663 | -0.08991 | NS | NS |
| Macrophages.M1 | T.cells.regulatory..Tregs. | 0.056663 | -0.08991 | NS | NS |
| T.cells.gamma.delta | Mast.cells.resting | 0.054839 | -0.09058 | NS | NS |
| Mast.cells.resting | T.cells.gamma.delta | 0.054839 | -0.09058 | NS | NS |
| T.cells.CD4.memory.activated | Monocytes | 0.052658 | -0.09141 | NS | NS |
| Monocytes | T.cells.CD4.memory.activated | 0.052658 | -0.09141 | NS | NS |
| T.cells.CD4.memory.activated | Mast.cells.activated | 0.050662 | -0.09219 | NS | NS |
| Mast.cells.activated | T.cells.CD4.memory.activated | 0.050662 | -0.09219 | NS | NS |
| T.cells.gamma.delta | Macrophages.M0 | 0.049026 | -0.09285 | Sig | Negative |
| Macrophages.M0 | T.cells.gamma.delta | 0.049026 | -0.09285 | Sig | Negative |
| T.cells.CD8 | Monocytes | 0.048827 | -0.09293 | Sig | Negative |
| Monocytes | T.cells.CD8 | 0.048827 | -0.09293 | Sig | Negative |
| NK.cells.activated | Eosinophils | 0.047083 | -0.09365 | Sig | Negative |
| Eosinophils | NK.cells.activated | 0.047083 | -0.09365 | Sig | Negative |
| Monocytes | B.cells.naive | 0.042505 | -0.09567 | Sig | Negative |
| B.cells.naive | Monocytes | 0.042505 | -0.09567 | Sig | Negative |
| T.cells.gamma.delta | Dendritic.cells.resting | 0.041989 | -0.09591 | Sig | Negative |
| Dendritic.cells.resting | T.cells.gamma.delta | 0.041989 | -0.09591 | Sig | Negative |
| Mast.cells.resting | B.cells.naive | 0.041388 | -0.09619 | Sig | Negative |
| B.cells.naive | Mast.cells.resting | 0.041388 | -0.09619 | Sig | Negative |
| Macrophages.M2 | Macrophages.M1 | 0.038759 | -0.09747 | Sig | Negative |
| Macrophages.M1 | Macrophages.M2 | 0.038759 | -0.09747 | Sig | Negative |
| Macrophages.M1 | B.cells.naive | 0.035867 | -0.09895 | Sig | Negative |
| B.cells.naive | Macrophages.M1 | 0.035867 | -0.09895 | Sig | Negative |
| T.cells.regulatory..Tregs. | Macrophages.M0 | 0.027786 | -0.10373 | Sig | Negative |
| Macrophages.M0 | T.cells.regulatory..Tregs. | 0.027786 | -0.10373 | Sig | Negative |
| T.cells.gamma.delta | B.cells.naive | 0.026039 | -0.10492 | Sig | Negative |
| B.cells.naive | T.cells.gamma.delta | 0.026039 | -0.10492 | Sig | Negative |
| Macrophages.M0 | B.cells.memory | 0.025539 | -0.10527 | Sig | Negative |
| B.cells.memory | Macrophages.M0 | 0.025539 | -0.10527 | Sig | Negative |
| T.cells.CD4.naive | NK.cells.activated | 0.0248 | -0.1058 | Sig | Negative |
| NK.cells.activated | T.cells.CD4.naive | 0.0248 | -0.1058 | Sig | Negative |
| T.cells.CD4.memory.activated | B.cells.naive | 0.020819 | -0.10893 | Sig | Negative |
| B.cells.naive | T.cells.CD4.memory.activated | 0.020819 | -0.10893 | Sig | Negative |
| T.cells.regulatory..Tregs. | Mast.cells.resting | 0.018313 | -0.11118 | Sig | Negative |
| Mast.cells.resting | T.cells.regulatory..Tregs. | 0.018313 | -0.11118 | Sig | Negative |
| T.cells.CD4.memory.activated | NK.cells.resting | 0.017625 | -0.11184 | Sig | Negative |
| NK.cells.resting | T.cells.CD4.memory.activated | 0.017625 | -0.11184 | Sig | Negative |
| T.cells.CD4.memory.activated | Eosinophils | 0.014343 | -0.11536 | Sig | Negative |
| Eosinophils | T.cells.CD4.memory.activated | 0.014343 | -0.11536 | Sig | Negative |
| T.cells.follicular.helper | Mast.cells.activated | 0.013234 | -0.11671 | Sig | Negative |
| Mast.cells.activated | T.cells.follicular.helper | 0.013234 | -0.11671 | Sig | Negative |
| T.cells.regulatory..Tregs. | Neutrophils | 0.012897 | -0.11714 | Sig | Negative |
| Neutrophils | T.cells.regulatory..Tregs. | 0.012897 | -0.11714 | Sig | Negative |
| T.cells.gamma.delta | NK.cells.resting | 0.011458 | -0.1191 | Sig | Negative |
| NK.cells.resting | T.cells.gamma.delta | 0.011458 | -0.1191 | Sig | Negative |
| NK.cells.activated | Macrophages.M2 | 0.009367 | -0.12237 | Sig | Negative |
| Macrophages.M2 | NK.cells.activated | 0.009367 | -0.12237 | Sig | Negative |
| T.cells.CD8 | Dendritic.cells.activated | 0.009043 | -0.12293 | Sig | Negative |
| Dendritic.cells.activated | T.cells.CD8 | 0.009043 | -0.12293 | Sig | Negative |
| T.cells.CD4.naive | Macrophages.M2 | 0.008517 | -0.12389 | Sig | Negative |
| Macrophages.M2 | T.cells.CD4.naive | 0.008517 | -0.12389 | Sig | Negative |
| Plasma.cells | Monocytes | 0.006843 | -0.12732 | Sig | Negative |
| Monocytes | Plasma.cells | 0.006843 | -0.12732 | Sig | Negative |
| Macrophages.M0 | B.cells.naive | 0.006382 | -0.1284 | Sig | Negative |
| B.cells.naive | Macrophages.M0 | 0.006382 | -0.1284 | Sig | Negative |
| T.cells.follicular.helper | T.cells.CD4.naive | 0.006042 | -0.12924 | Sig | Negative |
| T.cells.CD4.naive | T.cells.follicular.helper | 0.006042 | -0.12924 | Sig | Negative |
| Score | NK.cells.activated | 0.003675 | -0.13667 | Sig | Negative |
| NK.cells.activated | Score | 0.003675 | -0.13667 | Sig | Negative |
| NK.cells.resting | B.cells.naive | 0.003518 | -0.13731 | Sig | Negative |
| B.cells.naive | NK.cells.resting | 0.003518 | -0.13731 | Sig | Negative |
| T.cells.CD8 | T.cells.CD4.naive | 0.002049 | -0.14497 | Sig | Negative |
| T.cells.CD4.naive | T.cells.CD8 | 0.002049 | -0.14497 | Sig | Negative |
| T.cells.gamma.delta | Monocytes | 0.001439 | -0.14979 | Sig | Negative |
| Monocytes | T.cells.gamma.delta | 0.001439 | -0.14979 | Sig | Negative |
| T.cells.gamma.delta | Macrophages.M2 | 0.000816 | -0.15724 | Sig | Negative |
| Macrophages.M2 | T.cells.gamma.delta | 0.000816 | -0.15724 | Sig | Negative |
| T.cells.CD4.naive | Macrophages.M1 | 0.000808 | -0.15737 | Sig | Negative |
| Macrophages.M1 | T.cells.CD4.naive | 0.000808 | -0.15737 | Sig | Negative |
| T.cells.CD4.memory.resting | Macrophages.M1 | 0.000682 | -0.15954 | Sig | Negative |
| Macrophages.M1 | T.cells.CD4.memory.resting | 0.000682 | -0.15954 | Sig | Negative |
| T.cells.CD8 | Eosinophils | 0.000605 | -0.16105 | Sig | Negative |
| Eosinophils | T.cells.CD8 | 0.000605 | -0.16105 | Sig | Negative |
| T.cells.CD8 | Mast.cells.activated | 0.000419 | -0.16563 | Sig | Negative |
| Mast.cells.activated | T.cells.CD8 | 0.000419 | -0.16563 | Sig | Negative |
| T.cells.CD4.memory.activated | Mast.cells.resting | 0.000415 | -0.16573 | Sig | Negative |
| Mast.cells.resting | T.cells.CD4.memory.activated | 0.000415 | -0.16573 | Sig | Negative |
| T.cells.CD8 | B.cells.naive | 0.000275 | -0.17072 | Sig | Negative |
| B.cells.naive | T.cells.CD8 | 0.000275 | -0.17072 | Sig | Negative |
| Plasma.cells | Mast.cells.resting | 0.000269 | -0.17098 | Sig | Negative |
| Mast.cells.resting | Plasma.cells | 0.000269 | -0.17098 | Sig | Negative |
| NK.cells.activated | Mast.cells.activated | 0.000231 | -0.17275 | Sig | Negative |
| Mast.cells.activated | NK.cells.activated | 0.000231 | -0.17275 | Sig | Negative |
| Macrophages.M1 | Eosinophils | 0.000199 | -0.17453 | Sig | Negative |
| Eosinophils | Macrophages.M1 | 0.000199 | -0.17453 | Sig | Negative |
| Mast.cells.resting | Macrophages.M0 | 0.000156 | -0.17731 | Sig | Negative |
| Macrophages.M0 | Mast.cells.resting | 0.000156 | -0.17731 | Sig | Negative |
| T.cells.CD4.memory.resting | Plasma.cells | 0.000127 | -0.17969 | Sig | Negative |
| Plasma.cells | T.cells.CD4.memory.resting | 0.000127 | -0.17969 | Sig | Negative |
| Macrophages.M1 | Dendritic.cells.activated | 0.000103 | -0.18201 | Sig | Negative |
| Dendritic.cells.activated | Macrophages.M1 | 0.000103 | -0.18201 | Sig | Negative |
| Score | B.cells.memory | 8.59E-05 | -0.18407 | NS | NS |
| B.cells.memory | Score | 8.59E-05 | -0.18407 | NS | NS |
| Plasma.cells | NK.cells.resting | 6.57E-05 | -0.187 | NS | NS |
| NK.cells.resting | Plasma.cells | 6.57E-05 | -0.187 | NS | NS |
| T.cells.CD4.memory.activated | Macrophages.M2 | 4.73E-05 | -0.19058 | NS | NS |
| Macrophages.M2 | T.cells.CD4.memory.activated | 4.73E-05 | -0.19058 | NS | NS |
| Mast.cells.resting | Mast.cells.activated | 4.38E-05 | -0.19138 | NS | NS |
| Mast.cells.activated | Mast.cells.resting | 4.38E-05 | -0.19138 | NS | NS |
| T.cells.regulatory..Tregs. | T.cells.gamma.delta | 4.15E-05 | -0.19197 | NS | NS |
| T.cells.gamma.delta | T.cells.regulatory..Tregs. | 4.15E-05 | -0.19197 | NS | NS |
| Mast.cells.activated | Macrophages.M1 | 3.55E-05 | -0.19361 | NS | NS |
| Macrophages.M1 | Mast.cells.activated | 3.55E-05 | -0.19361 | NS | NS |
| T.cells.follicular.helper | T.cells.CD4.memory.resting | 6.00E-06 | -0.21154 | NS | NS |
| T.cells.CD4.memory.resting | T.cells.follicular.helper | 6.00E-06 | -0.21154 | NS | NS |
| Macrophages.M2 | Macrophages.M0 | 4.84E-06 | -0.21362 | NS | NS |
| Macrophages.M0 | Macrophages.M2 | 4.84E-06 | -0.21362 | NS | NS |
| Score | Plasma.cells | 4.63E-06 | -0.21403 | NS | NS |
| Plasma.cells | Score | 4.63E-06 | -0.21403 | NS | NS |
| Plasma.cells | Macrophages.M0 | 4.07E-06 | -0.21525 | NS | NS |
| Macrophages.M0 | Plasma.cells | 4.07E-06 | -0.21525 | NS | NS |
| Plasma.cells | Macrophages.M2 | 2.31E-06 | -0.22055 | NS | NS |
| Macrophages.M2 | Plasma.cells | 2.31E-06 | -0.22055 | NS | NS |
| Macrophages.M2 | B.cells.memory | 1.70E-06 | -0.22336 | NS | NS |
| B.cells.memory | Macrophages.M2 | 1.70E-06 | -0.22336 | NS | NS |
| Monocytes | Macrophages.M0 | 1.21E-06 | -0.22646 | NS | NS |
| Macrophages.M0 | Monocytes | 1.21E-06 | -0.22646 | NS | NS |
| Macrophages.M0 | Dendritic.cells.resting | 3.85E-07 | -0.23655 | NS | NS |
| Dendritic.cells.resting | Macrophages.M0 | 3.85E-07 | -0.23655 | NS | NS |
| T.cells.regulatory..Tregs. | T.cells.CD4.memory.activated | 2.93E-07 | -0.23889 | NS | NS |
| T.cells.CD4.memory.activated | T.cells.regulatory..Tregs. | 2.93E-07 | -0.23889 | NS | NS |
| T.cells.CD8 | Mast.cells.resting | 2.34E-07 | -0.24079 | NS | NS |
| Mast.cells.resting | T.cells.CD8 | 2.34E-07 | -0.24079 | NS | NS |
| T.cells.regulatory..Tregs. | Macrophages.M2 | 2.94E-08 | -0.25768 | NS | NS |
| Macrophages.M2 | T.cells.regulatory..Tregs. | 2.94E-08 | -0.25768 | NS | NS |
| T.cells.CD8 | NK.cells.resting | 3.49E-09 | -0.27386 | NS | NS |
| NK.cells.resting | T.cells.CD8 | 3.49E-09 | -0.27386 | NS | NS |
| T.cells.follicular.helper | Macrophages.M0 | 5.41E-10 | -0.2872 | NS | NS |
| Macrophages.M0 | T.cells.follicular.helper | 5.41E-10 | -0.2872 | NS | NS |
| T.cells.CD4.memory.activated | Macrophages.M0 | 4.27E-10 | -0.28884 | NS | NS |
| Macrophages.M0 | T.cells.CD4.memory.activated | 4.27E-10 | -0.28884 | NS | NS |
| NK.cells.resting | Macrophages.M1 | 4.09E-10 | -0.28914 | NS | NS |
| Macrophages.M1 | NK.cells.resting | 4.09E-10 | -0.28914 | NS | NS |
| T.cells.CD4.memory.resting | T.cells.CD4.memory.activated | 1.45E-10 | -0.29621 | NS | NS |
| T.cells.CD4.memory.activated | T.cells.CD4.memory.resting | 1.45E-10 | -0.29621 | NS | NS |
| NK.cells.activated | Macrophages.M0 | 2.11E-11 | -0.30887 | NS | NS |
| Macrophages.M0 | NK.cells.activated | 2.11E-11 | -0.30887 | NS | NS |
| T.cells.gamma.delta | Score | 9.98E-12 | -0.31362 | NS | NS |
| Score | T.cells.gamma.delta | 9.98E-12 | -0.31362 | NS | NS |
| NK.cells.resting | NK.cells.activated | 3.04E-12 | -0.32101 | NS | NS |
| NK.cells.activated | NK.cells.resting | 3.04E-12 | -0.32101 | NS | NS |
| T.cells.CD8 | Macrophages.M2 | 1.56E-12 | -0.32505 | NS | NS |
| Macrophages.M2 | T.cells.CD8 | 1.56E-12 | -0.32505 | NS | NS |
| Macrophages.M1 | Macrophages.M0 | 2.27E-18 | -0.39626 | NS | NS |
| Macrophages.M0 | Macrophages.M1 | 2.27E-18 | -0.39626 | NS | NS |
| Score | Macrophages.M1 | 3.39E-23 | -0.44432 | NS | NS |
| Macrophages.M1 | Score | 3.39E-23 | -0.44432 | NS | NS |
| T.cells.CD4.memory.activated | Score | 7.99E-24 | -0.45004 | NS | NS |
| Score | T.cells.CD4.memory.activated | 7.99E-24 | -0.45004 | NS | NS |
| T.cells.CD8 | T.cells.CD4.memory.resting | 1.68E-26 | -0.47331 | NS | NS |
| T.cells.CD4.memory.resting | T.cells.CD8 | 1.68E-26 | -0.47331 | NS | NS |
| T.cells.CD8 | Macrophages.M0 | 3.05E-29 | -0.4954 | NS | NS |
| Macrophages.M0 | T.cells.CD8 | 3.05E-29 | -0.4954 | NS | NS |
| T.cells.CD8 | Score | 8.99E-43 | -0.58559 | NS | NS |
| Score | T.cells.CD8 | 8.99E-43 | -0.58559 | NS | NS |

**NS:** No significant; **Sig:** significant.

**Supplementary Table S4. Prognostic analysis of immune cells.**

| **Immune cells** | **P value** | **HR** | **Change** |
| --- | --- | --- | --- |
| T.cells.CD8 | 0.000497841 | 0.620917893 | Favorable.factors |
| Macrophages.M1 | 0.001225606 | 0.643177714 | Favorable.factors |
| T.cells.CD4.memory.activated | 0.003286583 | 0.667500174 | Favorable.factors |
| Plasma.cells | 0.018792333 | 0.724261036 | Favorable.factors |
| Mast.cells.resting | 0.021816591 | 1.367831009 | Risk.factors |
| T.cells.CD4.memory.resting | 0.022792571 | 1.366289109 | Risk.factors |
| Mast.cells.activated | 0.040888357 | 1.688424325 | Risk.factors |
| NK.cells.resting | 0.051475162 | 1.310908754 | NS |
| Macrophages.M0 | 0.058393037 | 1.298036582 | NS |
| T.cells.gamma.delta | 0.060964054 | 0.769267254 | NS |
| Macrophages.M2 | 0.088553228 | 1.259504567 | NS |
| Eosinophils | 0.096931926 | 1.449425006 | NS |
| B.cells.naive | 0.213081526 | 1.186544125 | NS |
| T.cells.CD4.naive | 0.225102498 | 1.379066319 | NS |
| Dendritic.cells.activated | 0.232525535 | 1.318178071 | NS |
| NK.cells.activated | 0.330493044 | 0.875169334 | NS |
| Neutrophils | 0.40158369 | 1.162604341 | NS |
| Dendritic.cells.resting | 0.426141715 | 0.896805583 | NS |
| T.cells.regulatory..Tregs. | 0.461921266 | 0.904048639 | NS |
| Monocytes | 0.477668826 | 1.102076691 | NS |
| T.cells.follicular.helper | 0.885082297 | 0.980346854 | NS |
| B.cells.memory | 0.941683294 | 0.988377733 | NS |

**HR:** Hazard ratio; **NS:** No significant.
